# Organocatalyzed synthesis of fluorinated poly(aryl thioethers)

**DOI:** 10.1038/s41467-017-00186-3

**Published:** 2017-08-01

**Authors:** Nathaniel H. Park, Gabriel dos Passos Gomes, Mareva Fevre, Gavin O. Jones, Igor V. Alabugin, James L. Hedrick

**Affiliations:** 1 IBM Almaden Research Center, 650 Harry Road, San Jose, CA 95120 USA; 2 Department of Chemistry and Biochemistry, Florida State University, Tallahassee, FL 32310 USA

## Abstract

The preparation of high-performance fluorinated poly(aryl thioethers) has received little attention compared to the corresponding poly(aryl ethers), despite the excellent physical properties displayed by many polysulfides. Herein, we report a highly efficient route to fluorinated poly(aryl thioethers) via an organocatalyzed nucleophilic aromatic substitution of silyl-protected dithiols. This approach requires low catalyst loadings, proceeds rapidly at room temperature, and is effective for many different perfluorinated or highly activated aryl monomers. Computational investigations of the reaction mechanism reveal an unexpected, concerted S_N_Ar mechanism, with the organocatalyst playing a critical, dual-activation role in facilitating the process. Not only does this remarkable reactivity enable rapid access to fluorinated poly(aryl thioethers), but also opens new avenues for the processing, fabrication, and functionalization of fluorinated materials with easy removal of the volatile catalyst and TMSF byproducts.

## Abstract (web summary)

Fluorinated poly(aryl thioethers), unlike their poly(aryl ethers) counterparts, have received little attention despite excellent physical properties displayed by many polysulfides. Here the authors show a highly efficient route to fluorinated poly(aryl thioethers) via an organocatalyzed nucleophilic aromatic substitution of silyl-protected dithiols.

## Introduction

**Fig. 1:**
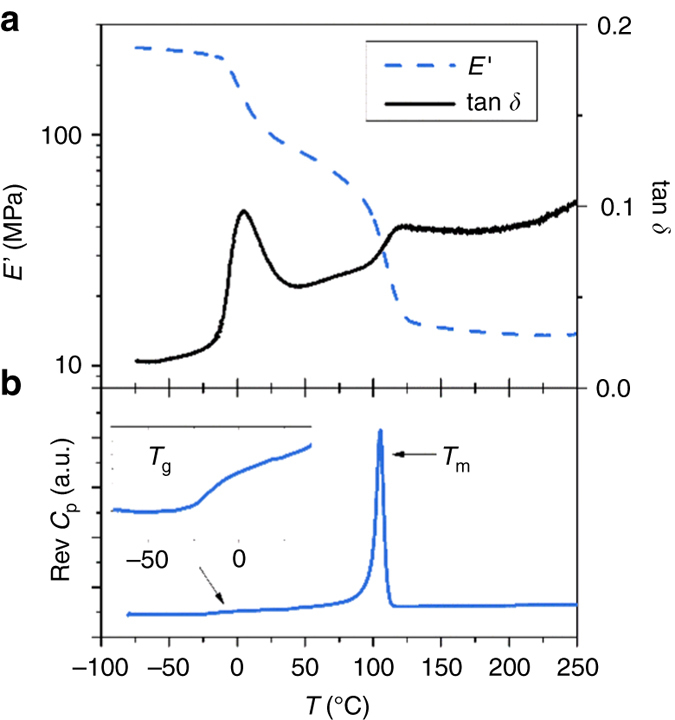
Thermal analysis of fluorinated poly(aryl thioethers). **a** DMA analysis of **1b**. **b** DSC analysis of **1b**. The sample used for DSC analysis was isolated by precipitation, while a braid for DMA was prepared with NMP-solutions of **1a** and hexafluorobenzene

Fluorinated materials exhibit many highly desirable properties such as increased chemical resistance, hydrophobicity, and thermal stability. One common route to introduce fluorine into polymer backbones is through the polycondensation of perfluoroarene-containing monomers (Fig. 1)^1–12^. These perfluorinated aromatic polymers impart increased order and stability to processed materials by forming energetically favorable π–π stacking arrangements with non-perfluoroarenes^13, 14^, as evidenced by their thermal properties^4^. Fluorinated aromatic polymers often possess a lower refractive index and optical loss than non-fluorinated analogs, making them excellent candidate materials for optical material applications^3, 5^. Given the advantages of having perfluoroarene units in polymers, we felt that the preparation of high performance fluorinated poly(aryl thioethers) would be ideally suited for use in coating and device applications.

As with perfluoroaryl-containing polymers, poly(aryl thioethers) are attractive materials that display excellent performance characteristics such as high thermal stability and hydrophobicity^15^. Poly(aryl thioethers) are traditionally prepared via nucleophilic aromatic substitution (S_N_Ar) under conditions that utilize stoichiometric amounts of base, extended reaction times, or in some cases, high temperatures^16–21^. Although these conditions have been utilized for the incorporation of perfluoroaryl-containing monomers into poly(aryl thioethers)^22^, they are not ideal, given the propensity of perfluoroarenes to undergo multiple substitutions^3, 23–26^ potentially leading to uncontrolled branching or cross-linking. Additionally, the stoichiometric amount of salt generated under standard S_N_Ar conditions could hamper further applications such as casting films to prepare hydrophobic surfaces. To circumvent the necessity of using stoichiometric base for the S_N_Ar reaction, we sought conditions for catalytic generation and reaction of thiolates. Here, we focused our attention on trimethylsilyl-protected thioethers, which could be cleaved by a catalyst to reveal a thiolate nucleophile. This intermediate could then undergo S_N_Ar with a perfluoroarene, thereby liberating trimethylsilylfluoride (TMSF) and regenerating the catalyst for subsequent reactions. Repetition of this cycle would give rise to the desired fluorinated poly(aryl thioether). Although similar protocols have been utilized for the preparation of polyethers^27, 28^, polysulfoxides^29^, polythiophenes^1, 5^, and poly(phenyleneethynylene)s^3^, there are no reports on the use of silylated dithiols as monomers. Thus, in order to take full advantage of the unique properties of fluoropolymers and poly(aryl thioethers), we developed a catalytic approach for the direct polymerization of perfluoroarenes into poly(aryl thioether) systems under mild conditions. This approach facilitates the polymerization of a variety of thiol nucleophiles and fluoroarenes and concurrent computational investigation of the reaction mechanism reveals a unique role of the catalyst in facilitating the polymerization process.

## Results

### Evaluation of polymerization catalysts

We began by preparing thioether **1a** (Table 1) as the trimethylsilane (TMS) protected nucleophile. By reacting **1a** with hexafluorobenzene using 5 mol % DBU as the organocatalyst^27^, a swift exotherm was observed, coupled with the formation of fluorotrimethylsilane (TMSF) and a rapid precipitation of polymeric material having a *M*_n_ of 8456 g/mol and a dispersity of 4.88 (entry 1, Table 1). The fast rate of reaction was consistent with preliminary time course experiments at higher catalyst loadings, which revealed the complete consumption of hexafluorobenzene within several seconds (see Supplementary Fig. 1). For further comparison of different catalyst systems and catalyst loadings, we selected 15 min as a benchmark reaction time. The large dispersity observed (entry 1, Table 1) may be the result of cross-linking or branching via multiple substitutions on the arene ring and would be consistent with the high reactivity of hexafluorobenzene^3, 23–26^. However, examination of the ^19^F NMR spectrum reveals a clean singlet indicative of a symmetrically substituted perfluoroarene ring and therefore the dispersity of the isolated material is more likely a result of kinetic quenching from the precipitation of the polymeric material. By lowering the catalyst loading to 1 or 0.5 mol %, the corresponding *M*_n_ and dispersity of the polymers decreased while still affording short reaction times (entries 2 and 3, Table 1). Performing the reaction inside a glovebox under strictly anhydrous conditions afforded a higher *M*_n_ and dispersity, indicating that the polymerization reaction is likely sensitive to water and ambient moisture (entry 4, Table 1).

**Table 1:** Evaluation of catalysts and catalyst loadings


Entry	Catalyst	Mol %	Time	${M}_{n}^{\rm a}$	${M}_{w}^{\rm a}$	*Ð* ^a^
1	DBU	5	15 min	8456	41 241	4.88
2	DBU	1	15 min	7128	22 597	3.17
3	DBU	0.5	15 min	6926	17 438	2.52
4^b^	DBU	0.5	15 min	33210	120 743	3.64
5	TBD	0.5	15 min	6017	24 047	4.00
6	DMC	0.5	15 min	6804	19 075	2.80
7	TBAF	0.5	15 min	7509	44 617	5.94
8^c^	Et_3_N	10	14 h	2455	4159	1.69
9^d^	*i*Pr_2_NEt	10	14 h	3043	5844	1.92
10^e^	DABCO	10	14 h	2750	4704	1.71
11^f^	*i*Pr_2_NEt	10	16 h	9267	22 868	2.47

Reagents and conditions: **1a** (0.25–0.5 mmol), hexafluorobenzene (0.25–0.5 mmol), catalyst (0.5–10 mol %), DMF (1 M), rt, 15 min–16 h. *DBU*: 1,8-diazobicyclo(5.4.0)undec-7-ene, *TBD*: triazabicyclodecene, *DMC: N,N*′-dicyclohexyl-4-morpholineformamidine, *TBAF*: tetra-n-butylammonium fluoride, *DABCO*: 1,4-diazabicyclo(2.2.2)octane

^a^Determined by SEC calibrated with polystyrene standards and using THF as the eluent

^b^Reaction run inside glovebox

^c^Reaction gave 94% conversion of hexafluorobenzene based on ^19^F analysis of crude reaction mixture using PhCF_3_ as an internal standard

^d^96% conversion of hexafluorobenzene

^e^98% conversion of hexafluorobenzene

^f^Reaction run at 100 °C

Other catalyst systems in addition to DBU were also evaluated. Guanidine containing catalysts such as TBD and DMC, gave similar results as compared to DBU (entries 5 and 6, Table 1). TBD afforded a higher dispersity, presumably due to increased basicity and hence reactivity. Less basic catalysts, such as triethylamine, DIEA, and DABCO, all gave polymers with a narrower dispersity than their more strongly basic counterparts (entries 8–10, Table 1). However, the corresponding molecular weights of the resultant polymers were lower and longer reaction times were required. By heating the reaction with 10 mol % of diisopropylethylamine; however, results similar to those using DBU could be obtained (entry 11, Table 1).

### Thermal properties of fluorinated poly(aryl thioethers)

The thermal properties **1b** were investigated by thermogravimetric analysis (TGA), differential scanning calorimetry (DSC) and dynamic mechanical analysis (DMA). The *T*_2%_ of **1b**, which was isolated by precipitation prior to analysis, was found at 333 °C (see Supplementary Fig. 2). Figure 1 shows the reverse Cp plot of the same isolated polymer (Fig. 1b), as well as DMA traces of in situ polymerized solutions of **1a** and C_6_F_6_ on a support braid (Fig. 1a) in NMP. Two thermal transitions were detected on the DSC thermogram in the studied temperature range: a glass transition around −18 °C and a melting endotherm around 105 °C, evidence of the semi-crystalline morphology of **1b** (Fig. 1b), consistent with other perfluoroarene-containing poly(aryl thioethers)^10, 22^. These data were in agreement with the DMA plots, where two E’ drops along with two tan δ maxima were observed (Fig. 1a).

### Evaluation of other monomers

Having investigated the thermal properties of **1a** and identified the appropriate catalyst systems and reaction parameters, we next utilized this approach to polymerize other fluoroarene electrophiles and silyl thioethers. Decafluorobiphenyl proved to be an excellent substrate for this reaction and readily polymerized when **1a** was used as the nucleophile with either DBU or TBD as the catalyst (**2b**, entries 1 and 2, Table 2). The TMS thioether of 4,4′-thiodibenzenethiol (**2a**, Table 2) was also a viable monomer for polymerization with perfluoroarenes. Interestingly, when decafluorobiphenyl was utilized as a co-monomer with **2a**, no catalyst was necessary as dissolution of both monomers in DMF was sufficient to induce rapid polymerization to afford **2c** (entry 3, Table 2). Presumably, this reactivity is due to the increased lability of TMS-protected aryl thioethers relative to TMS-protected alkyl thioethers. Both reactions to produce **2a** and **2b** (entries 1–3, Table 2) gave polymers that are comparable to those produced using unprotected thiols and stoichiometric base (see Supplementary Figs. 3 and 4), highlighting the efficacy of this approach to produce fluorinated poly(aryl thioethers) without stoichiometric salt byproducts. For synthesizing **2d**, a catalyst was still needed to facilitate the polymerization of **2a** and hexafluorobenzene (entry 4, Table 2). Non-perfluorinated, yet highly activated aryl electrophiles such as bis(4-fluoro-3-nitrophenyl) sulfone could also be rapidly polymerized under the reaction conditions to form **2e** (Table 2). Unfortunately, the very limited solubility of polymers **2d** and **2e** prevented their analysis via GPC or NMR, although the *T*_g_ for these materials could be obtained from DSC analysis (entries 4 and 5, Table 2).

**Table 2:** Evaluation of other monomers for the preparation of poly(aryl thioethers)


Entry	Poly.	Cat.	Mol %	${M}_{n}^{\rm a}$	${M}_{w}^{\rm a}$	*Ð* ^a^	T_g_ (°C)^b^	Yield^c^
1^d^	**2b**	TBD	1	9585	27 898	2.91	—	94%
2^e^	**2b**	DBU	1	15701	40 353	2.57	16.7	—
3^d,f^	**2c**	None	—	77 68	47 026	6.05	150.0	97%
4^f,g^	**2d**	DBU	0.25	—	—	—	48.0	75%
5^g^	**2e**	DBU	0.5	—	—	—	28.0	64%

Reagents and conditions: **1a** or **2a** (0.25–0.5 mmol), aryl electrophile (0.25–0.5 mmol), catalyst (0.25–1 mol %), DMF (1 M), rt, 5–15 min

^a^Determined by SEC using polystyrene standards and THF as the eluent

^b^Determined by DSC on the second heating cycle

^c^Based on mass of recovered material

^d^Reaction time was 5 min

^e^Reaction time was 15 min

^f^Reaction run inside glovebox

^g^Reaction time was 10 min

### Computational mechanistic investigation

**Fig. 2:**
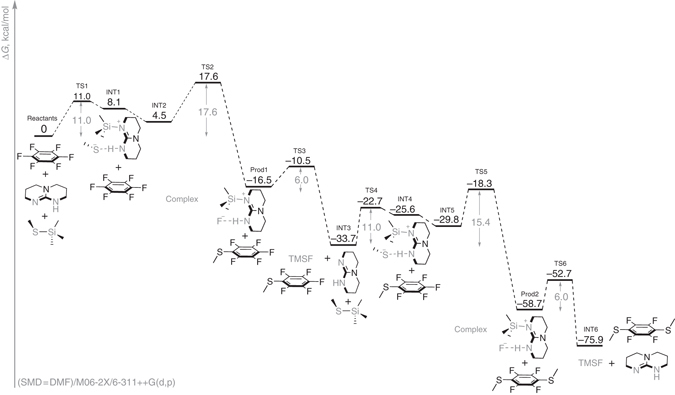
Computational analysis of the TBD-catalyzed reaction pathway. Free energy profile, structures and energetics for the first two steps in the TBD-catalyzed thioetherification of hexafluorobenzene by MeS−TMS

To understand the origins of the aforementioned remarkable reactivity, we performed computational investigations with the M06-2X density functional method on the mechanisms and energetics for the TBD-catalyzed reaction of hexafluorobenzene with TMS-protected methanethiol (TMS–SMe) (Fig. 2). The nucleophilic attack of TBD on the TMS group of TMS–SMe and displacement of methanethiolate (MeS^−^) from the silyl protecting group was identified as a key starting point. This process results in the formation of complex INT1 —where MeS^−^ is hydrogen-bonded to the TBD–TMS cation— and then associates with C_6_F_6_ to form the productive trimolecular complex INT2 (Fig. 2). The free energy of INT2 formation illustrates that strong enthalpic contributions of attractive supramolecular interactions largely compensate for the unfavorable entropy (ΔH = −8.4 kcal/mol, Δ*G* = + 4.5 kcal/mol relative to the separated components). Next, complex INT2 reacts to afford the mono-substituted product (Prod1) via TS2, in which TBD promotes the attack of MeS^−^ on the aromatic ring as F^-^ leaves (Fig. 2). This contrasts directly with the typical, stepwise addition-elimination mechanism of S_N_Ar reactions, as the TBD-catalyzed reaction proceeds in a concerted manner where formation of the C–S bond is coordinated with scission of the C–F bond^27, 30–32^. The TBD catalyst serves dual roles in the S_N_Ar process occurring in TS2: it delivers the MeS^−^ nucleophile and assists the concomitant displacement of the fluorine atom through a hydrogen bonding interaction (Fig. 2). The free energy barrier for the TBD-assisted thioetherification and fluoride displacement is only 17.6 kcal/mol, presumably owing to synergistic interactions present in the TS, which allows for the scission of a very strong C–F bond to proceed with such a small penalty.

**Fig. 3:**
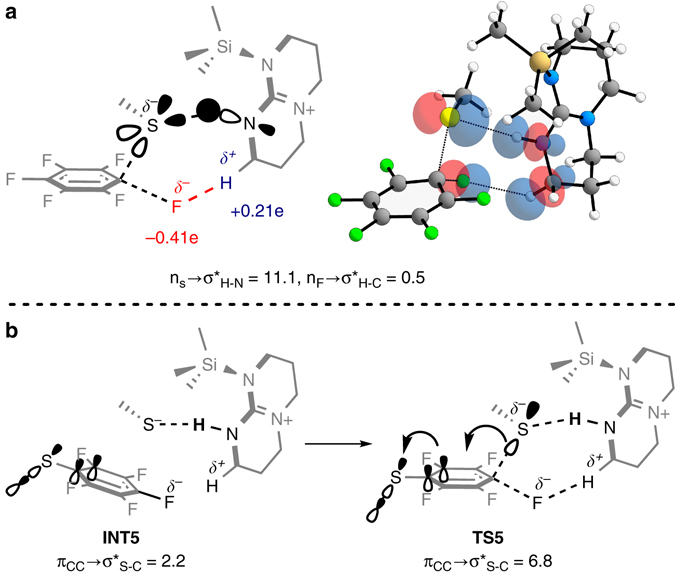
Computational analysis of transition state stabilization interactions. **a** NBO analysis of TS2. Highlighted interactions are responsible for stabilization of this TS. Second-order perturbation energies, in kcal/mol. **b** NBO analysis of INT5 and TS5. Highlighted interactions are responsible for stabilization of each structure. Second-order perturbation energies, in kcal/mol

These synergistic interactions include the covalent attachment of TBD to the TMS group, which renders the TBD catalyst cationic, and thereby enhances the hydrogen-bond interaction between the N–H group and the sulfide by n_S_→σ*_H−N_ donation due to the decreased electron density on the N–H proton (Fig. 3a; see Supplementary Fig. 5 for an S–H scan from the MeS^−^(TBD-TMS)^+^ complex). Furthermore, the relatively acidic α-CH bonds adjacent to the acceptor N–H moiety can provide stabilization to the departing fluoride via C–H•••F interactions (see Supplementary Figs. 6 and 7 for computed natural charges). Electrostatic stabilization due to such interactions has been reported to be significant, even in the absence of strong n_F_/σ*_C–H_ overlap^32^. Finally, the rigid nature of the catalyst leads to a unique stereoelectronic advantage, as the N–H•••S interaction is associated with the in-plane lone pairs of sulfur, leaving the out-of-plane p-type lone pair fully available for the nucleophilic attack at the aromatic π-system (Fig. 3a).

The TBD catalyst is regenerated in INT3 by attack of the hydrogen-bonded fluoride anion on the neighboring TMS group in a low-barrier process with a free energy barrier of 6.0 kcal/mol with reference to the low-lying Prod1 (Fig. 2). The catalyst re-enters the cycle by activating a protected thiol to form INT4 in a mechanism analogous to INT1 formation. Complexation of INT4 with MeSPhF_5_ results in the formation of INT5, a complex similar to INT2, in which the deprotected thiolate is poised for nucleophilic attack *para* to the thiol substituent (Fig. 2). The presence of SMe substituent in the aromatic ring lowers the free energy barrier by 2.2 kcal/mol, making the second substitution roughly ~33 times faster than without the SMe (TS5, Fig. 2; Supplementary Fig. 8). The activating effect of the SMe group originates from an unusual geometry at the aryl thioether junction, with the SMe substituent rotating to the near orthogonality out-of-plane of the aromatic ring. The conformational change converts the aryl thioether from a moderate p-donor into a moderate σ-acceptor (Fig. 3b)^33, 34^. This is shown in Fig. 3b, as the π_CC_→σ$_{{\rm{S}} - {\rm{C}}}^ *$ interaction increases from 2.2 to 6.8 kcal/mol (4.6 kcal/mol difference), delocalizing more of the electronic density in the TS and lowering the overall free energy barrier for TS5 (Fig. 2). Following TS5, the TBD catalyst is regenerated in a similar low-barrier process as before (TS6, Fig. 2) to give the final products TMSF, TBD, and the *para*-disubstituted fluoroarene (INT6, Fig. 2).

## Discussion

Overall, we have developed an organocatalyzed reaction for the synthesis of fluorinated and non-fluorinated poly(aryl thioethers). As opposed to standard S_N_Ar reactions between thiols and perfluoroarenes, our conditions avoid the generation of stoichiometric salt by-products. This advantage, in combination with short reaction times, room temperature conditions, and low to no catalyst loadings, will enable new routes for processing the prepared fluoropolymers into devices and coatings. Computational examination of the mechanistic underpinnings of this process reveals a unique, concerted mechanism for the S_N_Ar reaction between silyl protected thioethers and perfluoroarenes, where the organocatalyst plays a critical, dual-activation role. Future work will endeavor to leverage these mechanistic and synthetic insights to further refine control over the polymerization reaction conditions for the development of both new fluorinated materials and seamless material processing techniques with minimal byproduct generation.

## Methods

### General procedure for polymer synthesis

An 8 ml screw-cap vial equipped with a magnetic stir-bar was charged with the thioether monomer (1 equiv) and fluoroarene (1−1.05 equiv), if solid. Solvent was then added, followed by any monomer that is a liquid (thioether or fluoroarene). The reaction mixture was then stirred to fully mix and dissolve the monomers. The catalyst (0.5–10 mol %) was then added and the reaction mixture was stirred for the indicated time at the specified temperature. Following completion of the reaction, methanol (8 ml) was added to precipitate the polymer. The solid was collected via centrifugation and decanting of the supernatant. Additional methanol (8 ml) was added to the recovered solid and the process centrifugation and decanting was repeated a second time. After drying, the isolated sample was analyzed via NMR and GPC. See Supplementary Figs. for NMR spectra.

### Synthesis of **1b**

In a nitrogen-filled glovebox and in accordance with the general procedure, a mixture of **1a** (84 µl, 0.25 mmol), hexafluorobenzene (28 µl, 0.25 mmol), DBU (20 µl of a 0.062 M stock solution in DMF), and DMF (0.25 ml) were stirred at room temperature for 15 min. After 15 min, the vial was removed from the glovebox and subjected to the workup in accordance with the general procedure, affording the polymer as a white solid. *M*_n_ = 33210 g/mol, *M*_w_ = 120743 g/mol, *Ð* = 3.64. ^1^H NMR (400 MHz, CDCl_3_) *δ* 2.92 (m, 4 H), 1.56 (m, 4 H), 1.41 (m, 4 H). ^19^F NMR (128 MHz, CDCl_3_) −135.07. *T*_g_ (DSC): −18 °C.

### Synthesis of **2b**

In accordance with the general procedure, a mixture of **1a** (161 µl, 0.48 mmol), decafluorobiphenyl (167 mg, 0.5 mmol), DBU (0.75 µl, 0.005 mmol; added as a stock solution in 0.1 ml DMF), and DMF (0.5 ml) were stirred at room temperature for 15 min. Following the workup in accordance with the general procedure, the polymer was isolated as a white solid. *M*_n_ = 15701 g/mol, *M*_w_ = 40353 g/mol, *Ð* = 2.57. ^1^H NMR (400 MHz, CDCl_3_) *δ* 3.03 (m, 4 H), 1.65 (m, 4 H), 1.48 (m, 4 H). ^19^F NMR (128 MHz, CDCl_3_) *δ* −134.6 (m, 4 F), −139.2 (m, 4 F). *T*_g_ (DSC): 16.7 °C. For the analogous TBD catalyzed polymerization the mass recovered was 209 mg (94%).

### Synthesis of **2c**

In a nitrogen filled glovebox and in accordance with the general procedure, a mixture of **2a** (197 mg, 0.50 mmol), decafluorobiphenyl (168 ml, 0.50 mmol), and DMF (0.5 mmol) were stirred at room temperature for 5 min. Following the workup in accordance with the general procedure, the polymer was isolated as a white solid (mass recovered: 263 mg, 94%). Note: Polymer retained residual DMF after workup and drying. *M*_n_ = 7768 g/mol, *M*_w_ = 47026 g/mol, *Ð* = 6.05. ^1^H NMR (400 MHz, CDCl_3_) *δ* 7.34 (m, 8 H). ^19^F NMR (128 MHz, CDCl_3_) *δ* −132.8 (m, 4 F), −133.0 (m, 0.5 F), −137.9 (m, 4 F), −138.1 (m, 0.5 F). *T*_g_ (DSC): 150 °C.

### Synthesis of **2d**

In a nitrogen-filled glovebox and in accordance with the general procedure, a mixture of **2a** (197 mg, 0.50 mmol), hexafluorobenzene (56 µl, 0.50 mmol), DBU (20 µl of a 0.062 M stock solution in DMF), and DMF (0.50 ml) were stirred at room temperature for 10 min. Following the workup in accordance with the general procedure, the polymer was isolated as a white solid (mass recovered: 218 mg, 75%). *T*_g_ (DSC): 48 °C. Note: The low solubility of the resulting polymer prevented full analysis by GPC or NMR.

### Synthesis of **2e**

In accordance with the general procedure, a mixture of **1a** (168 µl, 0.50 mmol), bis(4-fluoro-3-nitrophenyl)sulfone (172 mg, 0.50 mmol), DBU (20 µl of a 0.125 M stock solution in DMF), and DMF (0.5 ml) were stirred at room temperature for the indicated amount of time. Following the workup in accordance with the general procedure, the polymer was isolated as a light yellow solid (mass recovered: 145 mg, 64%). *T*_g_ (DSC): 28 °C. Note: The low solubility of the resulting polymer prevented full analysis by GPC or NMR.

### Data availability

All the data are available from authors upon reasonable request.

## Electronic supplementary material

Supplementary Information

Peer Review
