# Erratum: Organocatalyzed synthesis of fluorinated poly(aryl thioethers)

**DOI:** 10.1038/s41467-017-01129-8

**Published:** 2017-11-13

**Authors:** Nathaniel H. Park, Gabriel dos Passos Gomes, Mareva Fevre, Gavin O. Jones, Igor V. Alabugin, James L. Hedrick

**Affiliations:** 1 IBM Almaden Research Center, 650 Harry Road, San Jose, CA 95120 USA; 2 Department of Chemistry and Biochemistry, Florida State University, Tallahassee, FL 32310 USA

*Nature Communications* **8**:166 10.1038/s41467-017-00186-3; Article pubilshed online: 1 Aug 2017

Figure 2 of this article contains errors. In Fig. 2, the 3D structures of the molecules are not shown. The corrected version of Fig. 2 is shown below as Fig. 1.

**Figure 1:**
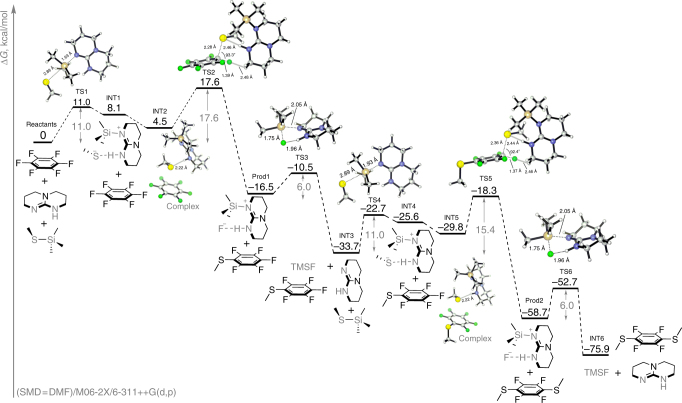
**Fig. 1**

