# Supplementary material for: Organocatalyzed synthesis of fluorinated poly(aryl thioethers)
Source: Nat Commun. 2017 Aug 1;8:166. doi: 10.1038/s41467-017-00186-3 (PMC5537313; doi:10.1038/s41467-017-00186-3)
Supplement: Supplementary file 1 — Supplementary Information [file 41467_2017_186_MOESM1_ESM.pdf]

File Name: Supplementary Information
Description: Supplementary Figures, Supplementary Methods and Supplementary References

File Name: Peer Review File
Description:

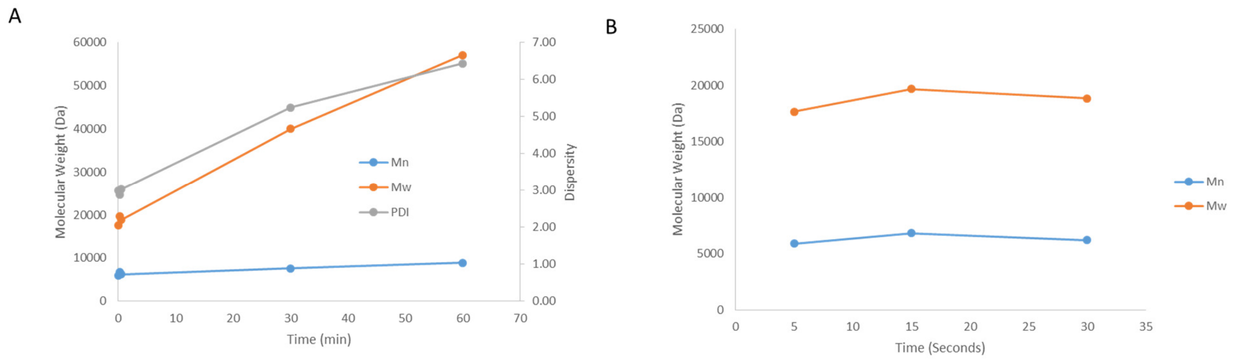

Entry	Conversion	Time	Mn	Mw	PDI
1	98%	5 s	5885	17652	3.00
2	99%	15 s	6828	19683	2.88
3	100%	30 s	6219	18850	3.03
4	100%	30 min	7628	40037	5.25
5	100%	60 min	8860	57078	6.44

Supplementary Figure 1: Time course study between the reaction of hexafluorobenzene and **1a** using 10 mol % DBU as a catalyst. A) Plot of M_n, M_w, and PDI versus time. B) Enlargement of first three time points. Conversion refers to conversion of hexafluorobenzene as determined by ^{19}F NMR analysis of crude reaction mixture using $PhCF_3$ as an internal standard.

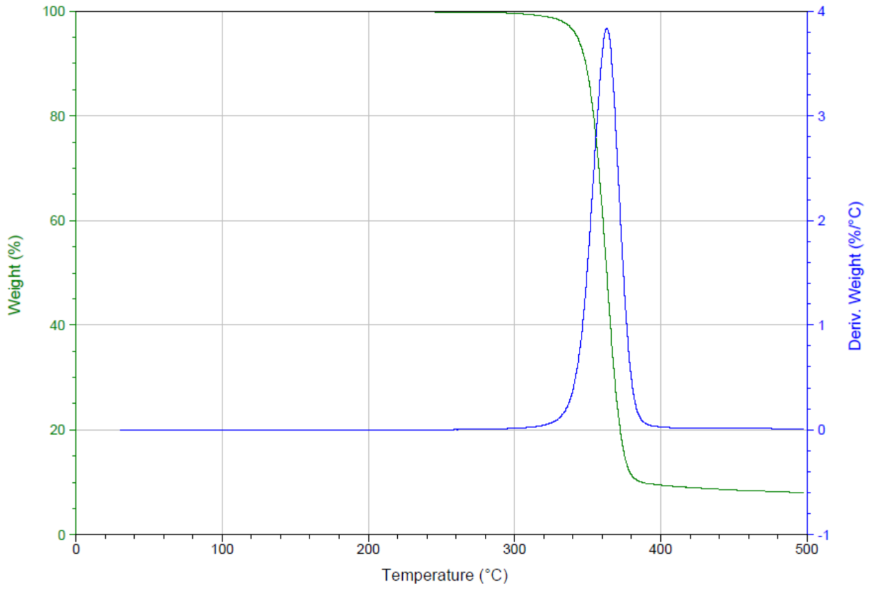

Supplementary Figure 2: TGA of polymer **1b**.

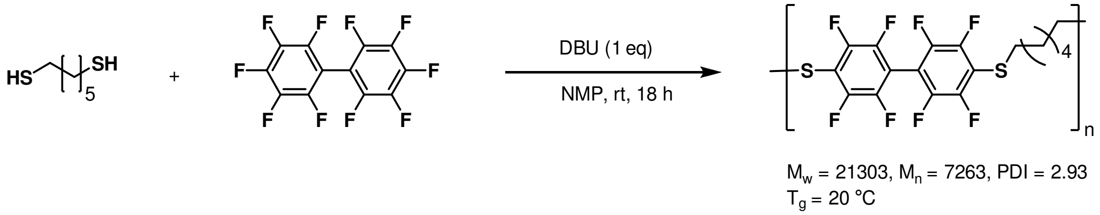

M_w = 21303, M_n = 7263, PDI = 2.93
T_g = 20 °C

Supplementary Figure 3: Polymerization of 1,6-hexanedithiol with decafluorobiphenyl using stoichiometric base.

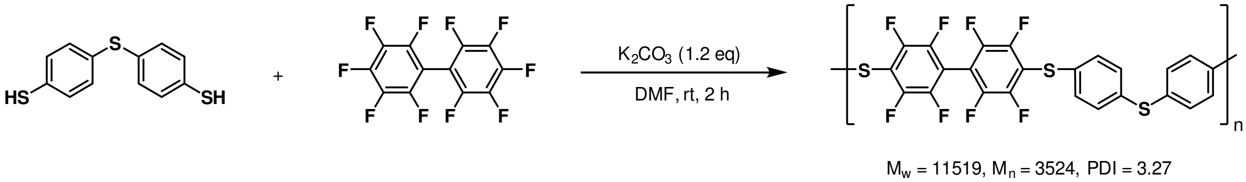

M_w = 11519, M_n = 3524, PDI = 3.27

Supplementary Figure 4: Polymerization of 4,4'-thiobisbenzenethiol with decafluorobiphenyl using stoichiometric base.

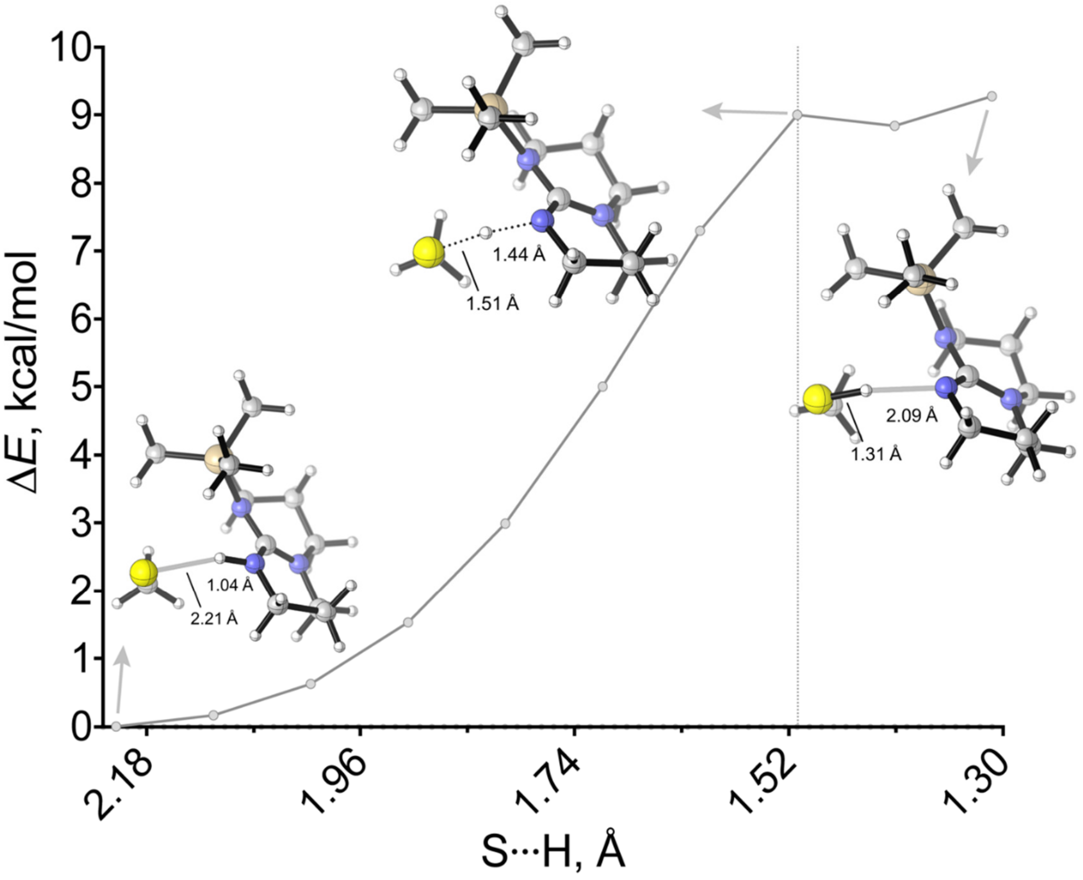

Supplementary Figure 5: S-H scan for formation of MeSH and TBD-TMS from the $MeS^{-}(TBD\text{-}TMS)^{+}$ complex.

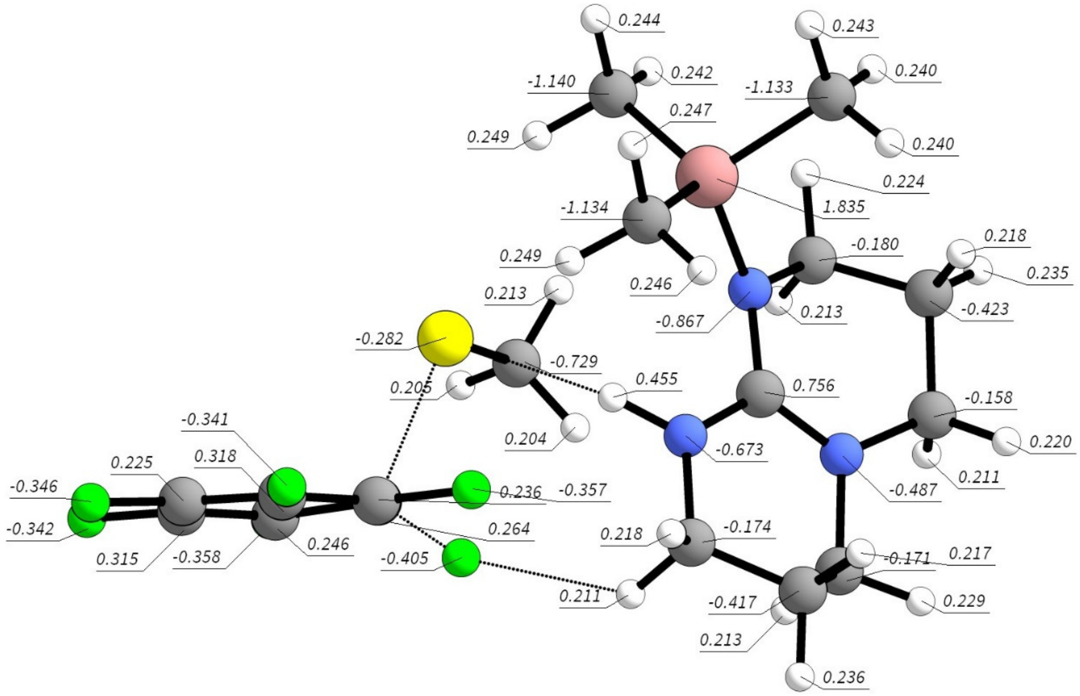

Supplementary Figure 6: Natural charges on TS2.

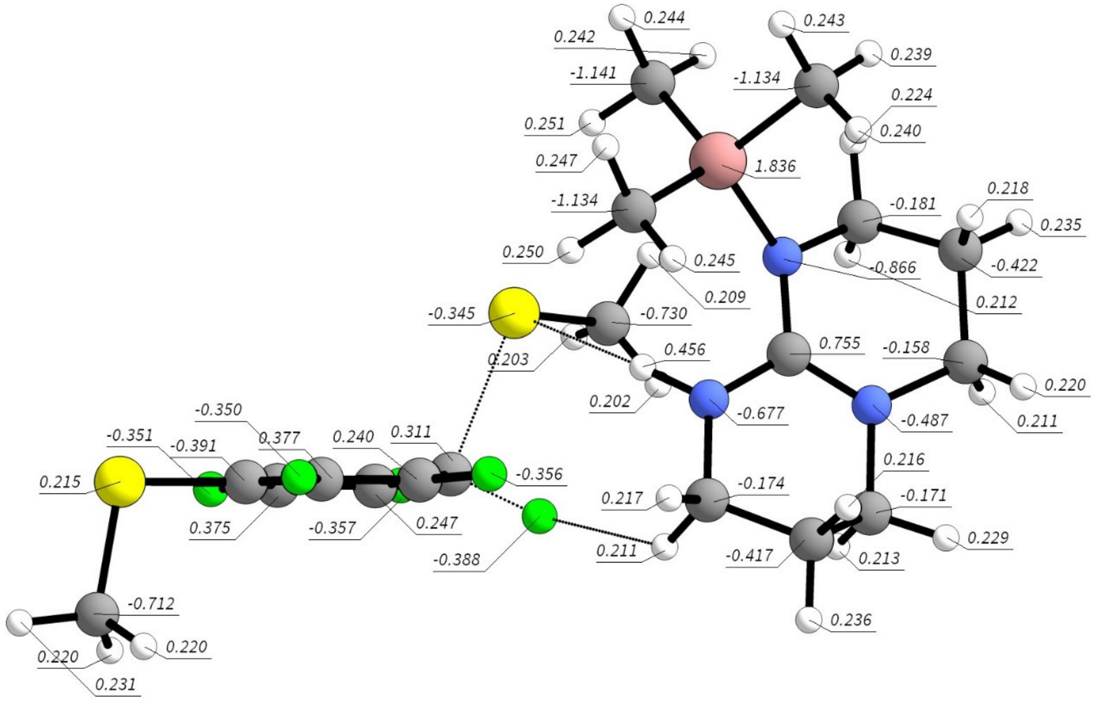

Supplementary Figure 7: Natural charges on TS5.

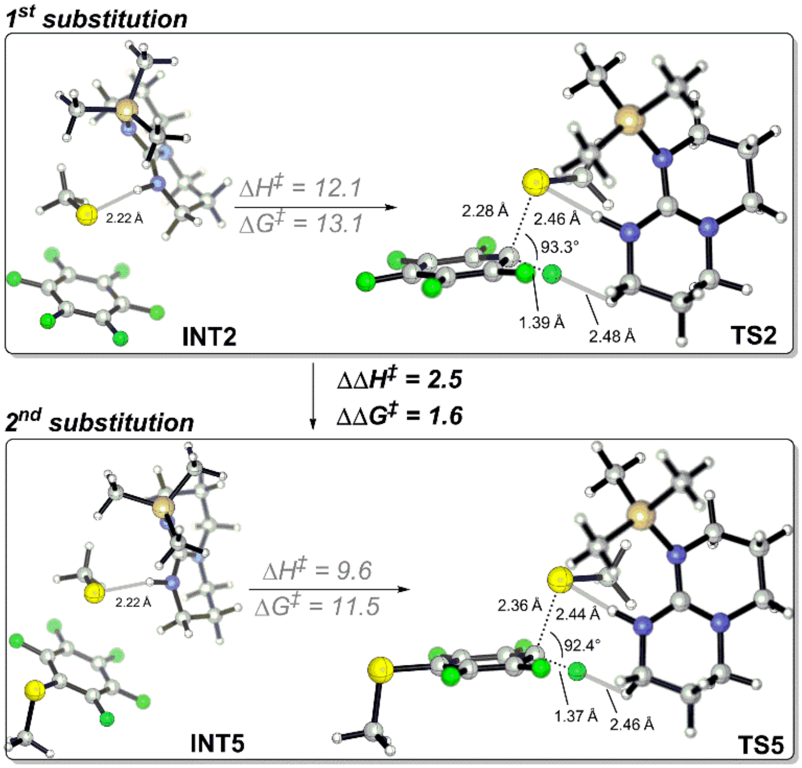

Supplementary Figure 8: Transition structures, enthalpies, and free energies for TBD-catalyzed methyl sulfide attack on PhF_6 and $MeSPhF_5$. Energies in kcal/mol. $\Delta\Delta H^{\ddagger}$ and $\Delta\Delta G^{\ddagger}$ values describe the difference between the 1st and the 2nd substitution barriers.

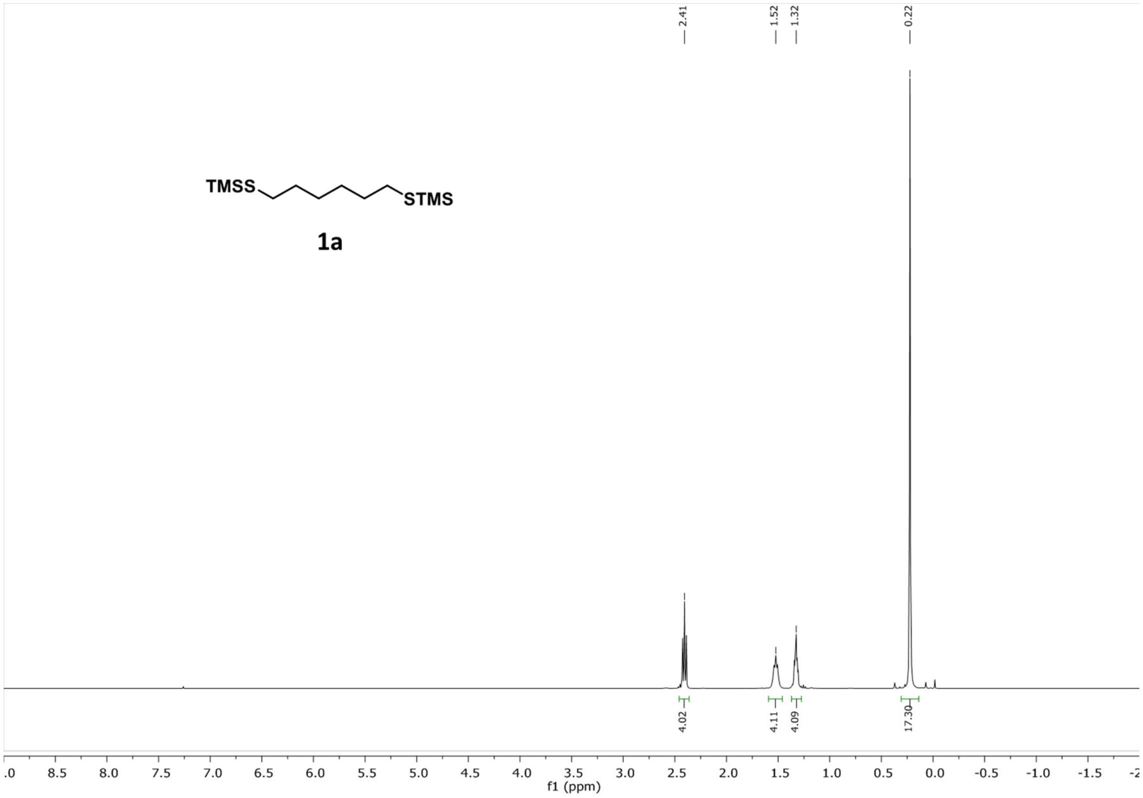

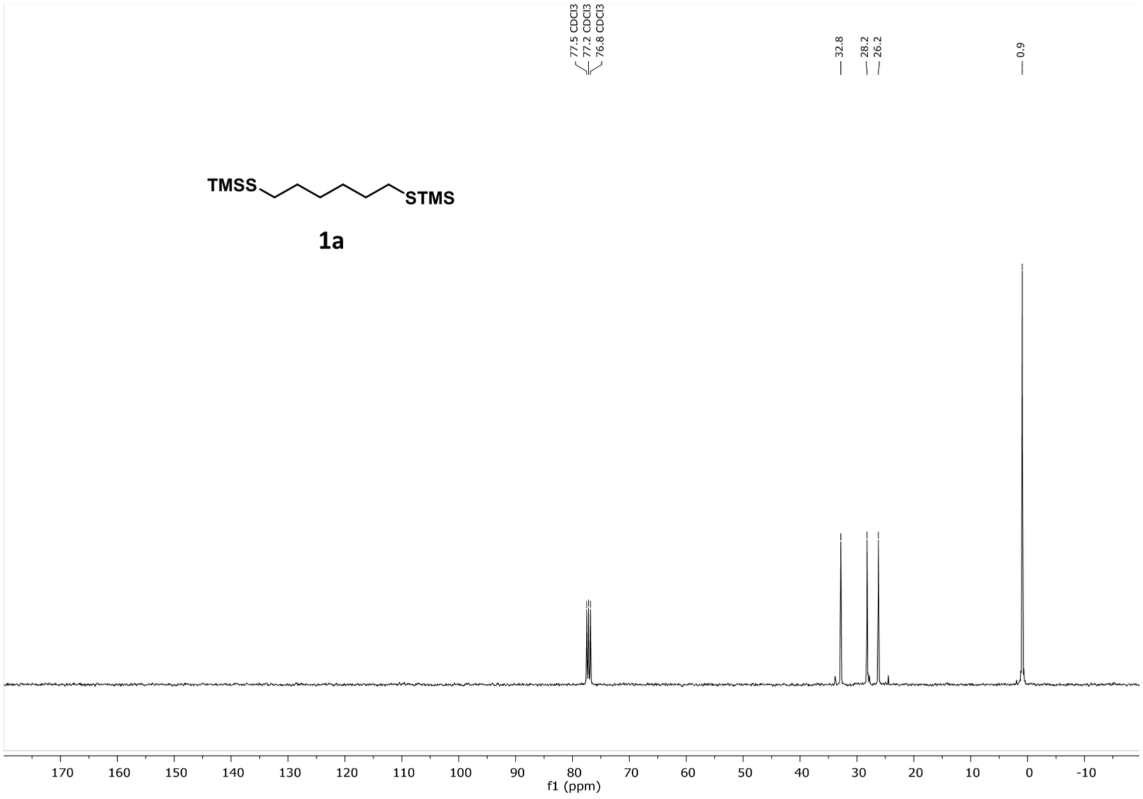

Supplementary Figure 9. ^{1}H NMR (top) and ^{13}C NMR (bottom) spectra of **1a** in $CDCl_3$.

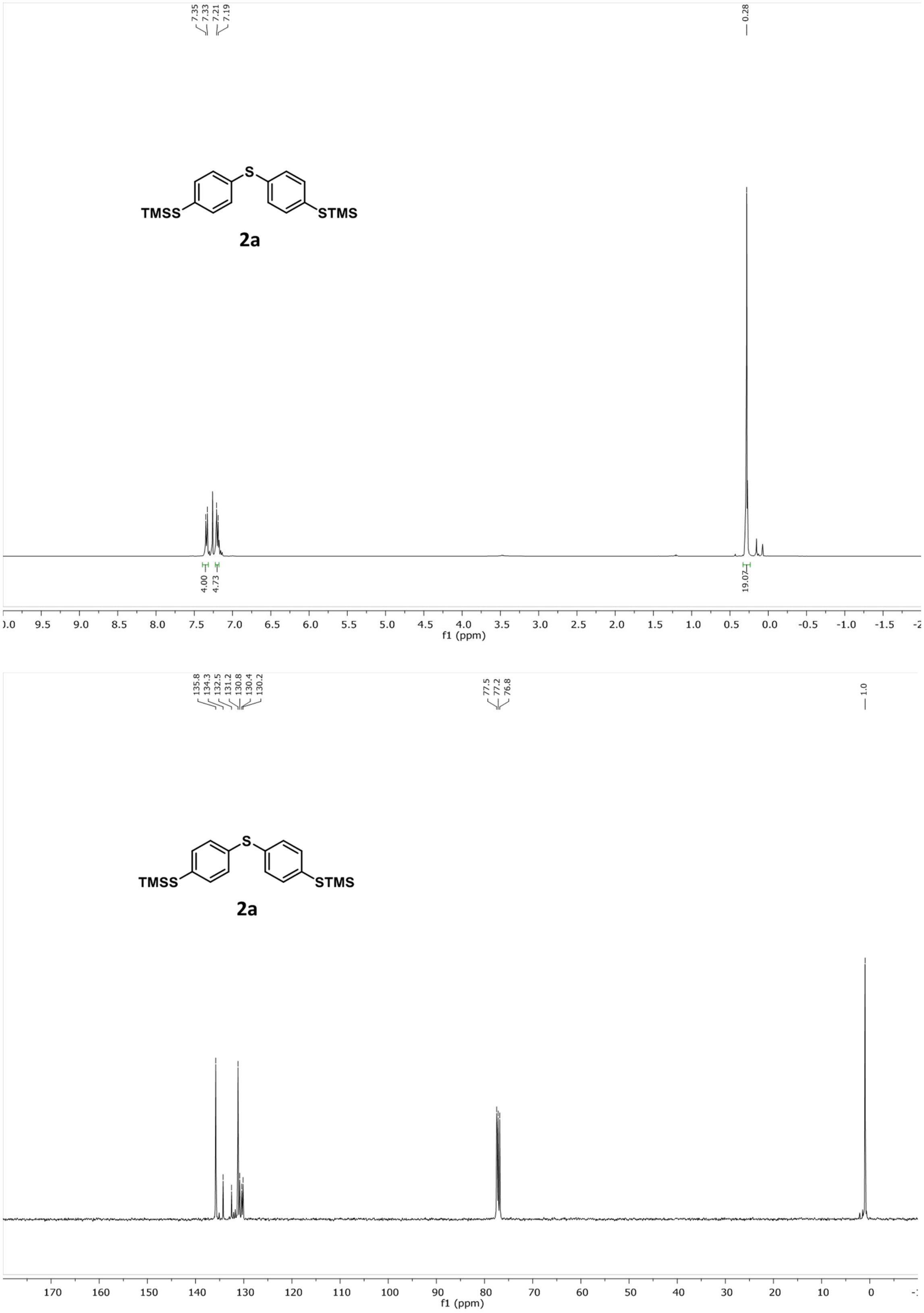

Supplementary Figure 10. ^{1}H NMR (top) and ^{13}C NMR (bottom) spectra of **2a** in $CDCl_3$.

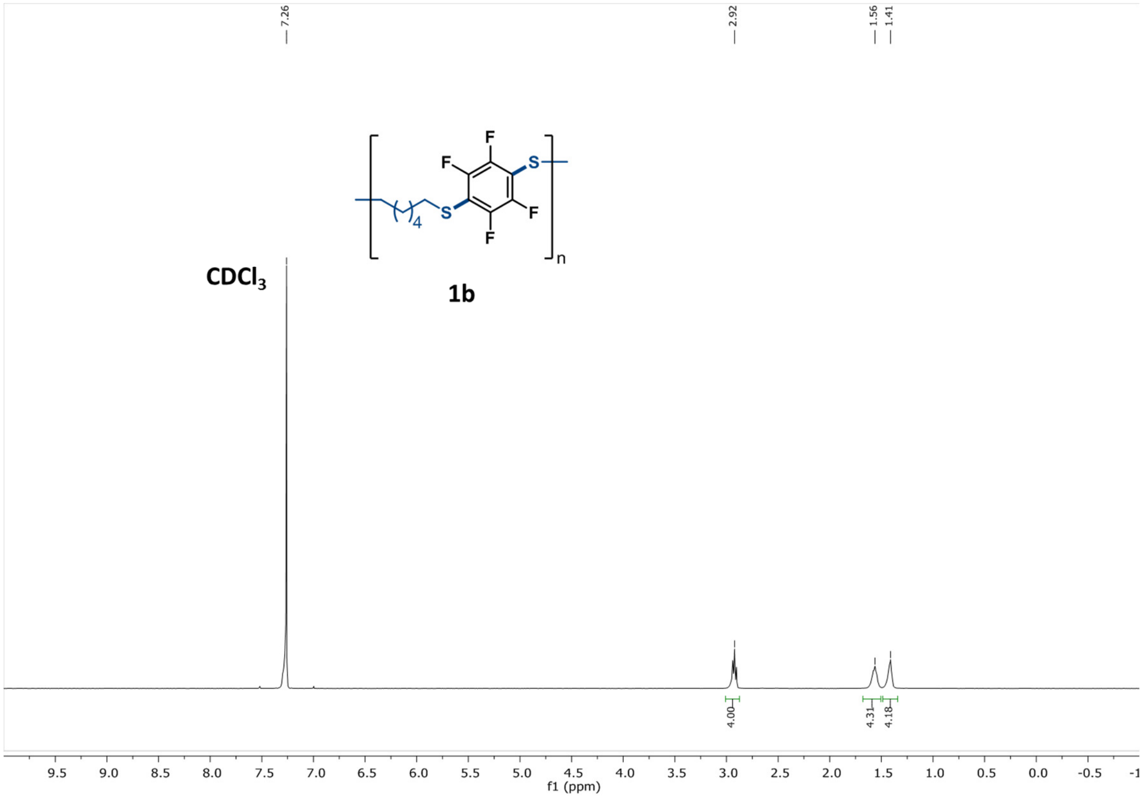

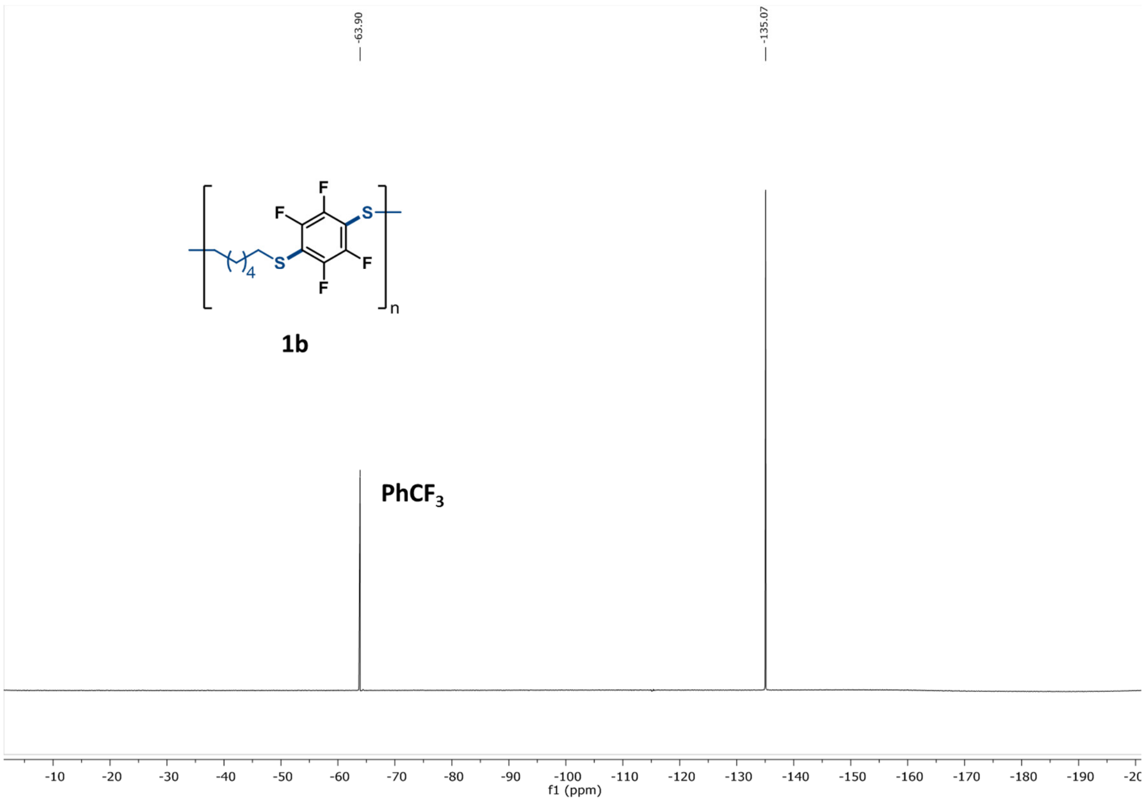

Supplementary Figure 11. ^{1}H NMR (top) and ^{19}F NMR (bottom) spectra of **1b** in $CDCl_3$.

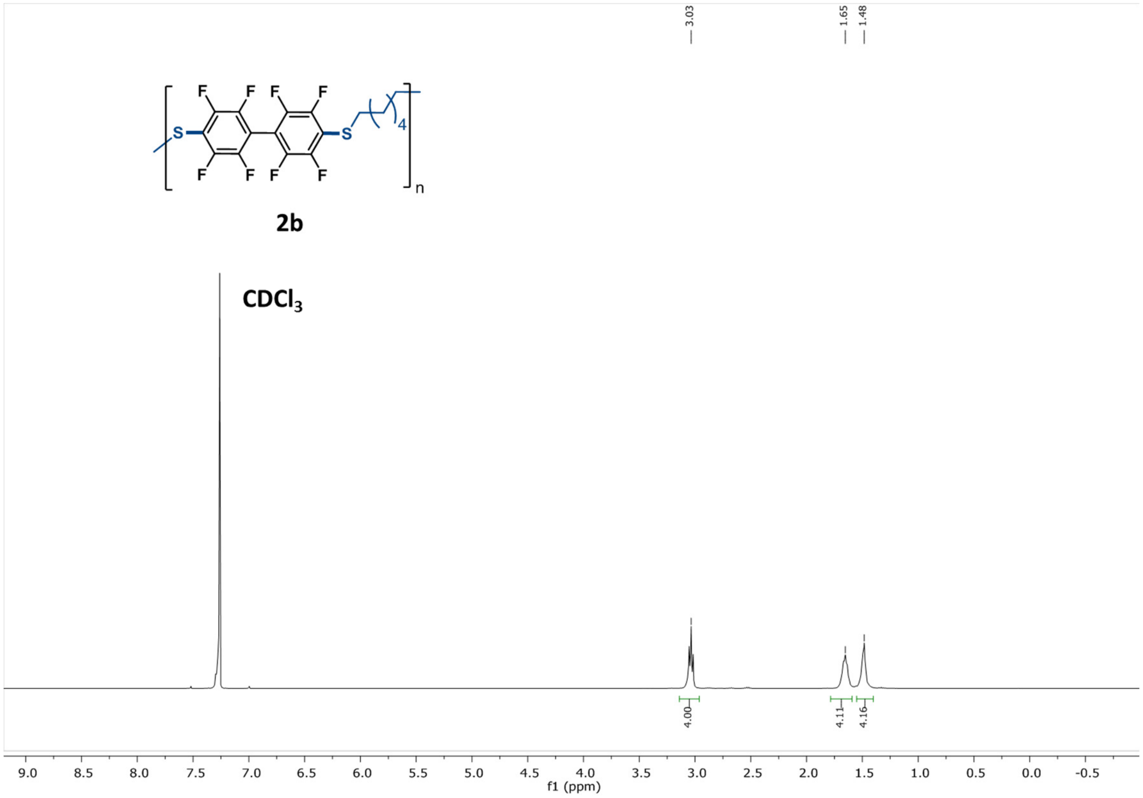

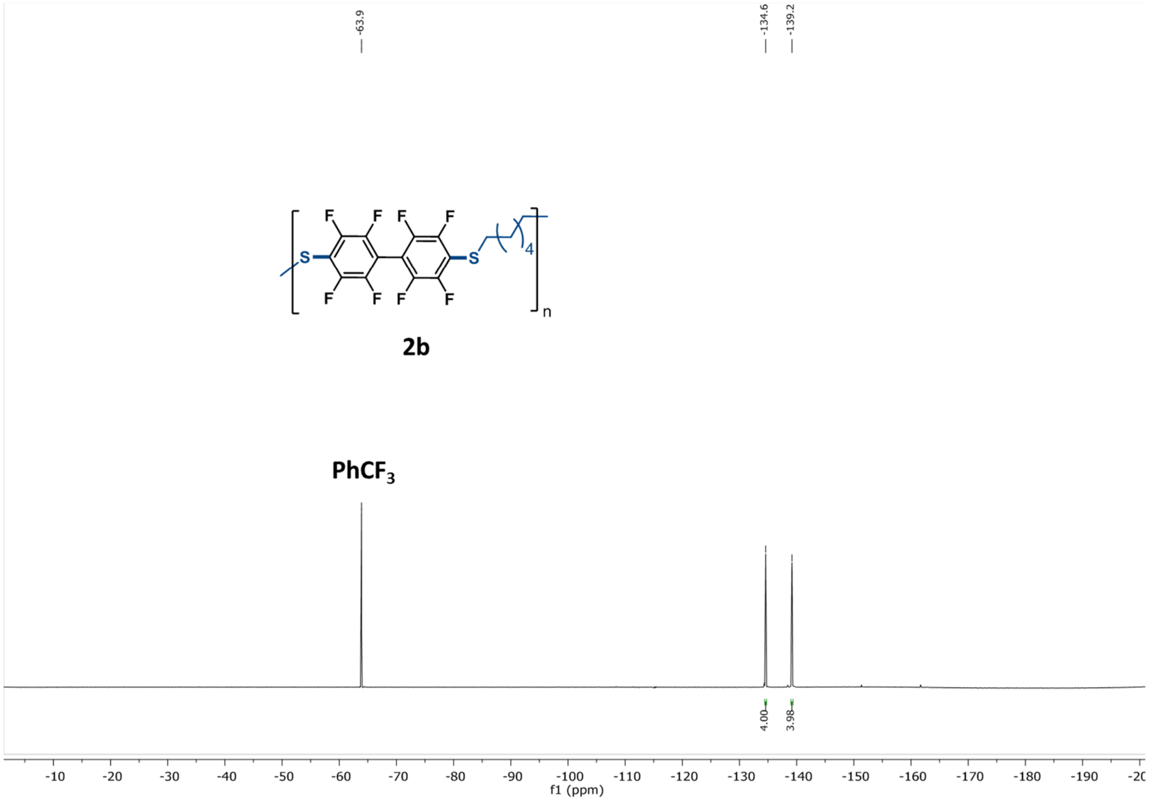

Supplementary Figure 12. ^{1}H NMR (top) and ^{19}F NMR (bottom) spectra of **2b** in $CDCl_3$.

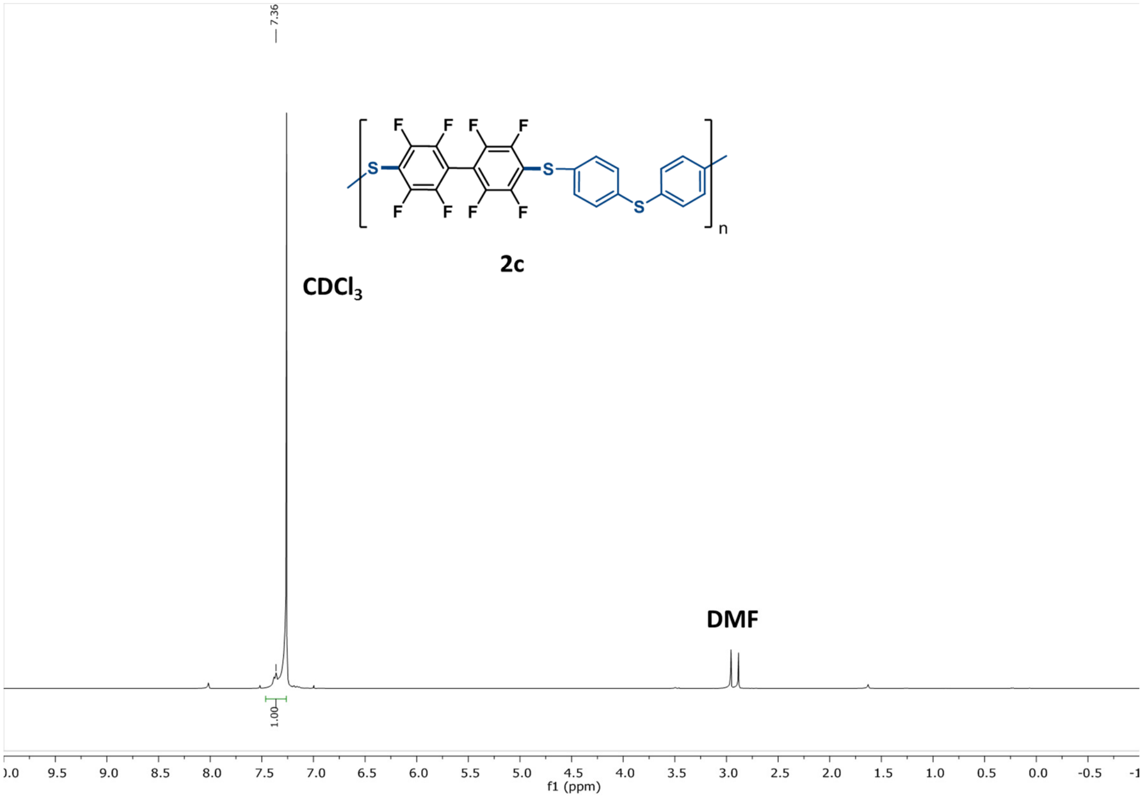

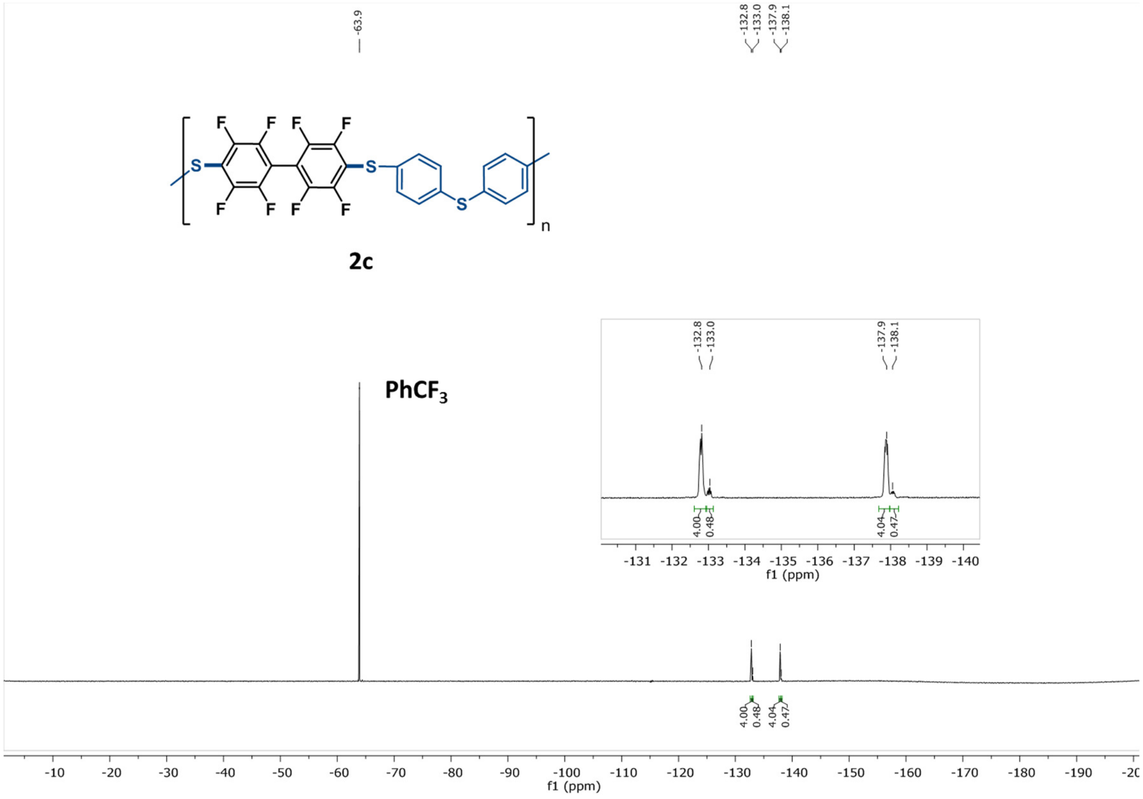

Supplementary Figure 13. ^{1}H NMR (top) and ^{19}F NMR (bottom) spectra of **2c** in $CDCl_3$.

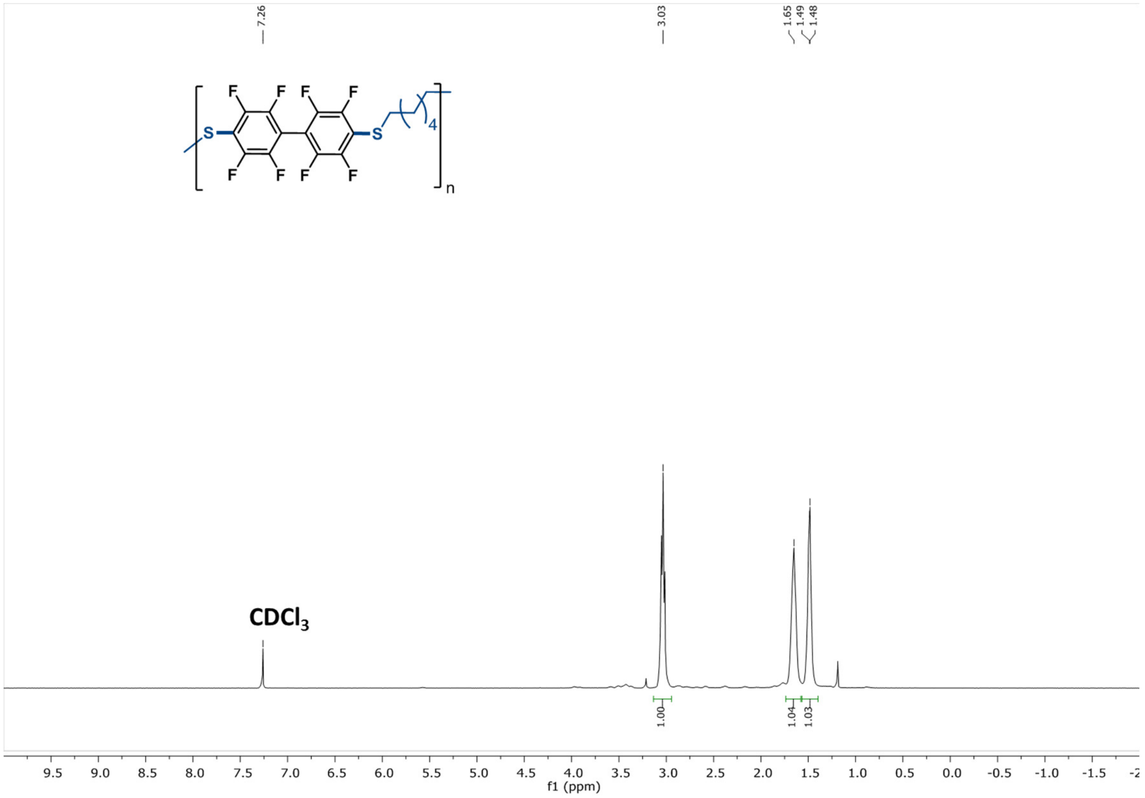

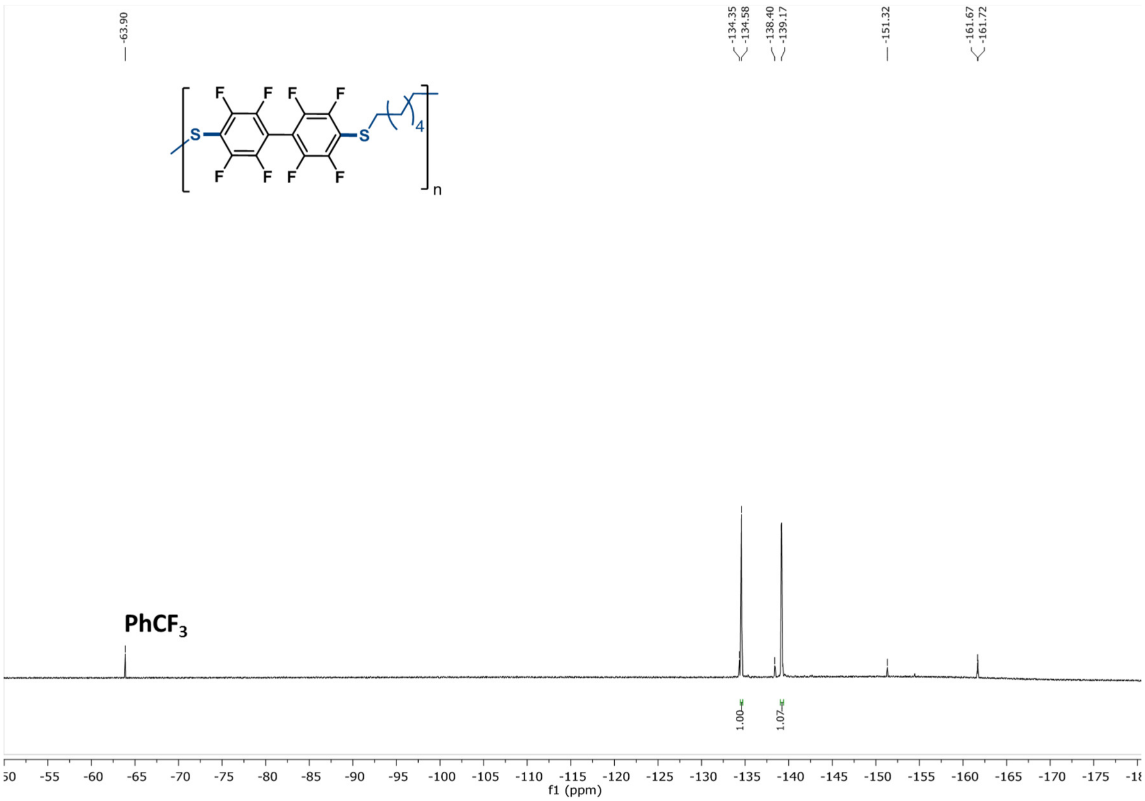

Supplementary Figure 14. ^{1}H NMR (top) and ^{19}F NMR (bottom) spectra of polymer for Figure S3.

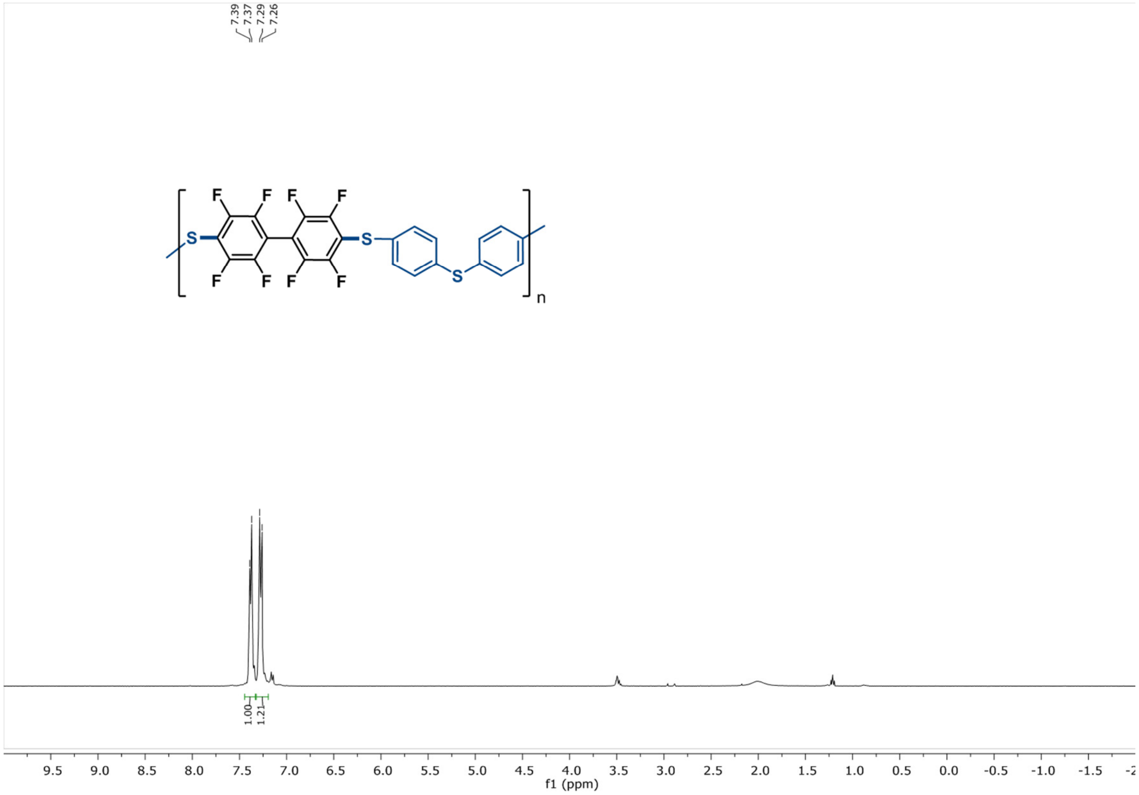

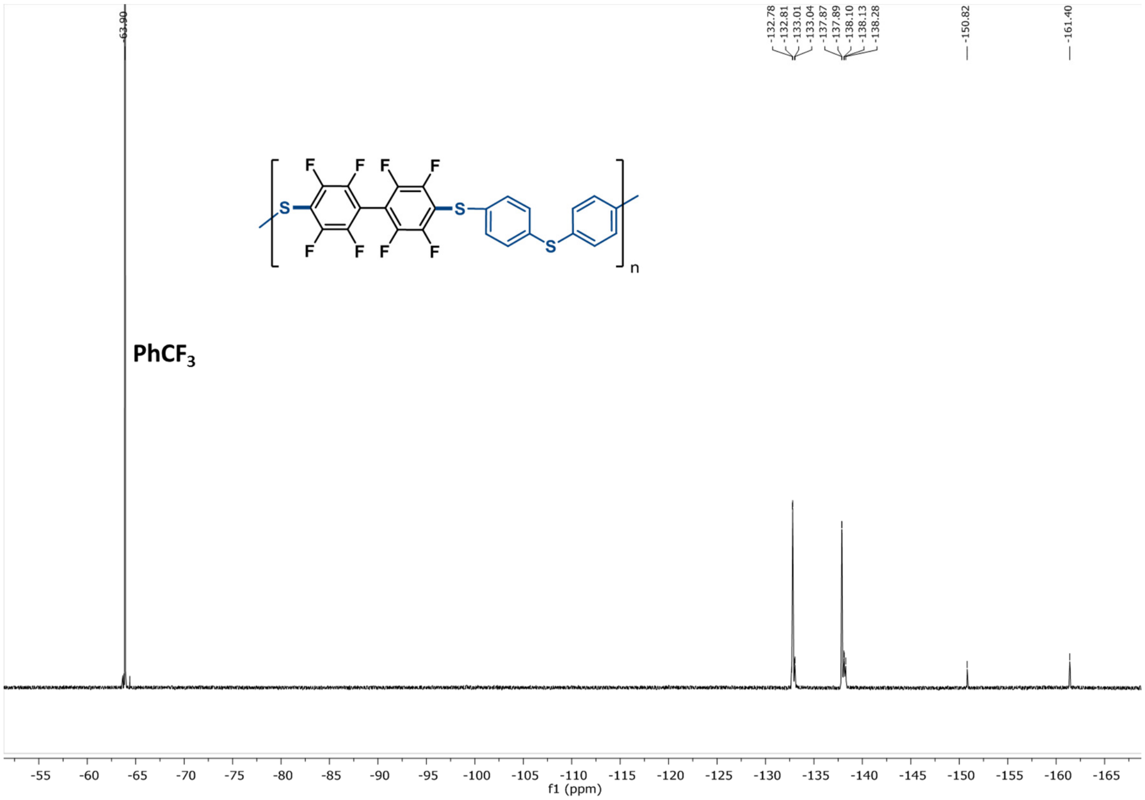

Supplementary Figure 15. ^{1}H NMR (top) and ^{19}F NMR (bottom) spectra of polymer for Figure S4.

Supplementary Methods:

General Reagent and Reaction Information. All reactions were performed on the benchtop with benchtop solvents under an atmosphere of air unless otherwise noted. All thioether monomers were stored in a nitrogen-filled glove box. Small amounts (~500 mg) were removed and stored in a vacuum desiccator with calcium sulfate as the desiccant for benchtop experiments. DMF for glovebox experiments was passed through two packed alumina columns under nitrogen and then stored in the glovebox over freshly activated 3 Å molecular sieves. All other reagents and solvents were commercially available and were used as received.

General Analytical Information. The ^{1}H NMRs of the polymers were recorded using a Bruker Avance 400 spectrometer, operated at 400 MHz with the solvent proton signal as the internal reference. SEC was conducted using THF (1.0 mL/min) as the eluent for monitoring the polymer conversion. THF-SEC was recorded on a Waters 2695D (Waters Corporation, USA) Separation Module equipped with an Optilab rEX differential refractometer (Wyatt Technology Corporation, U.S.A.) and Waters HR-4E as well as HR-1 columns (Waters Corporation, USA). Polymer solutions were prepared at a known concentration (ca. 3 mg/mL) and an injection volume of 100 µL was used. Data collection and analysis were performed using the Astra software (Wyatt Technology Corporation, USA; version 5.3.4.20). The columns were calibrated with series of polystyrene standards ranging from Mp = 360 Da to Mp = 778 kDa (Polymer Standard Service, USA). Thermogravimetric analyses (TGA) were performed on a TA Instruments Q500. The samples (7 to 9 mg of polymer isolated by precipitation) were scanned from RT to 500 °C at a 5 °C/min heating rate under an N_2 atmosphere. Differential scanning calorimetry (DSC) analyses were performed on a TA Instruments Q2000. The samples (7 to 9 mg of polymer isolated by precipitation) were scanned at a 5 °C/min heating rate in aluminum closed pans. The samples were subjected to a -80 ºC to 300 °C scan. Dynamic Mechanical Analyses (DMA) were performed on a TA Instruments DMA 2980 using a dual cantilever. The samples (approx. 12*6*1 mm) were prepared by coating braids with NMP solutions of **1a** and hexafluorobenzene, which were quickly mixed before deposition and curing at 220 °C for 1h to eliminate the NMP solvent and reaction byproducts. The braids were solicited from -80 ºC to 300 °C at a 5 °C/min heating rate twice and the reported trace corresponds to the second scan (first and second scans showed similar results max. +/- 8°C for the tan δ maxima).

Synthesis of monomers:

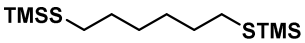

2,2,11,11-tetramethyl-3,10-dithia-2,11-disiladodecane (1a): A 500 mL round bottom flask under an atmosphere of nitrogen equipped with a magnetic stir-bar was charged with 1,6-hexanedithiol (2.5 mL, 22.3 mmol), Et_3N (6.2 mL, 49.2 mmol), and hexane (90 mL). TMSCl (6.9 mL,

49.2 mmol) was added via syringe and the reaction mixture was allowed to stir for 18 h at rt. After 18 h, the solid was removed via filtration and the filtrate was concentrated with the aid of a rotary evaporator. Additional hexane (100 mL) was added to the residue and the mixture was filtered again to remove any residual solid. The filtrate was concentrated with the aid of the rotary evaporator to provide the desired product as a colorless oil (4.54 g, 69%). ^{1}H NMR (400 MHz, $CDCl_3$) δ 2.41 (m, 4 H), 1.52 (m, 4 H), 1.32 (m, 4 H), 0.22 (s, 18 H). ^{13}C NMR (100 MHz, $CDCl_3$) δ 32.8, 28.2, 26.2, 0.9.

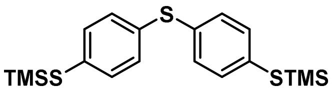

bis(4-((trimethylsilyl)thio)phenyl)sulfane (2a): A 500 mL round bottom flask under an atmosphere of nitrogen equipped with a magnetic stir-bar was charged was charged with 4,4'-thiodibenzenethiol (5.0 g, 20 mmol), Et_2O (100 mL), and CH_2Cl_2 (40 mL). TMSCl (5.6 mL, 44 mmol) was added followed by Et_3N (6.1 mL, 44 mmol) and the reaction mixture was stirred at rt. After stirring at rt for 18 h, the solid was removed by filtration and the filtrate was concentrated with the aid of a rotary evaporator. The residue was dissolved in Et_2O (150 mL), filtered, and concentrated to give the desired product as an off-white solid (6.6 g, 84%). *Note: The compound was used without further purification due to the sensitivity of the compound to moisture. As a result, the ^{13}C NMR was observed to contain residual impurities, likely due to small amounts of decomposition of the title compound.* ^{1}H NMR (400 MHz, $CDCl_3$) δ 7.44-7.42 (m, 4 H), 7.30-7.28 (m, 4 H), 0.37 (s, 18 H). ^{13}C NMR (100 MHz, $CDCl_3$) δ 135.8, 134.3, 132.5, 131.2, 130.8, 130.4, 130.2, 1.0.

Experimental Procedures:

Procedure for catalyst-free control experiment: In a nitrogen-filled glovebox, an 8 mL screw-cap vial equipped with a magnetic stir-bar was charged with **1a** (84 µL, 0.25 mmol), hexafluorobenze (28 µL, 0.25 mmol), and DMF (0.25 mL). The reaction mixture was stirred for 40 min at room temperature. Following completion of the reaction, methanol was added (8 mL) and no polymer precipitated. $PhCF_3$ (31 µL, 0.25 mmol) was added and an aliquot was removed for ^{19}F NMR analysis, which indicated no consumption of the hexafluorobenzene.

Procedure for preparation of braids for DMA: A screw-cap vial was charged with **1a** (229 µL, 0.68 mmol), hexafluorobenzene (80 µL, 0.71 mmol), and NMP (1.5 mL). A separate vial was charged with DBU (10.6 µL, 0.071 mmol) and NMP (0.5 mL). The catalyst and monomer solutions were mixed and the braid was saturated with the mixture. The saturated braid was heated to 60 °C for 1 h then cured at 220 °C for 4 h. A control braid was prepared in the same manner using a previously isolated polymer (prepared using the same procedure as **1a**) in an NMP solution.

Procedure for Supplementary Figure 1: A separate reaction was conducted for each individual time point. The general procedure for the reaction is as follows. A vial equipped with a magnetic stir-bar was charged with **1a** (84 μL, 0.25 mmol), hexafluorobenze (28 μL, 0.25 mmol), and DMF (0.25 mL). DBU (3.9 μL, 0.025 mmol) was added and the reaction mixture was stirred for the indicated time. After the completion of the indicated time, the reaction mixture was diluted with MeOH (8 mL) and $PhCF_3$ (31 μL, 0.25 mmol) was added. An aliquot was removed and analyzed by ^{19}F NMR to determine the conversion of hexafluorobenzene. The precipitated polymer was collected via centrifugation following decanting of the supernatant. After washing with additional MeOH (8 mL) and drying under vacuum, the recovered polymer was analyzed by GPC.

Procedure for Supplementary Figure 3: A vial equipped with a magnetic stir-bar was charged with decafluorobiphenyl (334 mg, 1.0 mmol) and NMP (1 mL) and stirred at room temperature until all solids had dissolved. Added 1,6-hexanedithiol (112 μL, 1.0 mmol) followed by DBU (300 μL, 2.0 mmol) and stirred the reaction mixture for 18 h at room temperature. After 18 h, MeOH (8 mL) was added and the precipitated polymer was collected via centrifugation. The isolated yellow solid was washed with additional MeOH (8 mL) and Et_2O (16 mL) and then dried under vacuum. M_n = 7263 g/mol, M_w = 21303 g/mol, $Đ$ = 2.93. ^1H NMR (400 MHz, $CDCl_3$) δ 3.03 (m, 2H), 1.65, (m, 2H), 1.48 (m, 2H). ^{19}F NMR (128 MHz, $CDCl_3$) δ -134.6 (m, 4F), -139.2 (m, 4F). T_g (DSC): 20 °C.

Procedure for Supplementary Figure 4: A vial equipped with a magnetic stir-bar was charged with decafluorobiphenyl (334 mg, 1.0 mmol), 4,4'-thiobisbenzenethiol (250 mg, 1.0 mmol), and DMF (1 mL) and stirred at room temperature for 2 h at room temperature. After 2 h, MeOH (8 mL) was added and the precipitated polymer was collected via centrifugation. The white solid was washed water (2 x 8 mL) and with additional MeOH (2 x 8 mL) and then dried under vacuum. M_n = 3524 g/mol, M_w = 11519 g/mol, $Đ$ = 3.27. ^1H NMR (400 MHz, $CDCl_3$) δ 7.39 (d, J = 7.8 Hz, 4H), 7.29 (d, J = 7.8 Hz, 4H). ^{19}F NMR (128 MHz, $CDCl_3$) δ -132.8 (m, 4F), -137.9 (m, 4F).

Computational Details

All calculations were performed using the software package *Gaussian '09*.[1] All geometry optimizations and NBO analysis were performed at the (SMD[2]=DMF)/M06-2X[3,4]/6-311++G(d,p) level of theory. The minimum energy pathway (MEP) was traced using the intrinsic reaction coordinate (IRC) method to ensure that each TS structure correctly links two minima. Frequency calculations were carried for all structures to confirm them as either a minimum or a TS. Electronic structures and properties were analyzed by the Natural Bond Orbitals (NBO)[5-9] method at the M06-2X level of theory. Three-dimensional structures were produced with both CYLView 1.0.1[10] and Chemcraft 1.8.

Calculated Geometries and Energies

Calculated geometries with their respective energies (in hartree) and number of imaginary frequencies.

C_6F_6

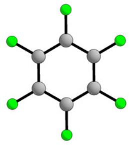

# of imaginary frequencies: 0

E = -827.597920332

C	1.137321000	-0.788765000	-0.000003000
C	-0.114363000	-1.379555000	0.000000000
C	-1.251648000	-0.590613000	0.000004000
C	-1.137321000	0.788765000	-0.000007000
C	0.114363000	1.379554000	-0.000003000
C	1.251647000	0.590613000	-0.000005000
F	2.228597000	-1.544910000	-0.000014000
F	2.452465000	1.156831000	0.000014000
F	0.224210000	2.702573000	0.000007000
F	-2.228597000	1.544910000	-0.000016000
F	-2.452466000	-1.156829000	0.000015000
F	-0.224209000	-2.702574000	0.000003000

TBD

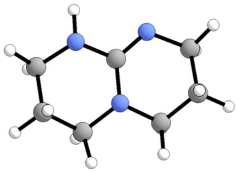

# of imaginary frequencies: 0

E = -438.775452963

N	-0.024738000	0.650824000	0.078972000

C	-0.060494000	-0.727856000	0.034610000
N	-1.120057000	-1.458245000	-0.068933000
C	-2.421022000	-0.807388000	-0.178644000
C	-2.437231000	0.608145000	0.383350000
C	-1.262605000	1.371501000	-0.199756000
H	-3.158904000	-1.426898000	0.339021000
H	-2.725682000	-0.781062000	-1.233319000
H	-2.343845000	0.580987000	1.473505000
H	-3.373583000	1.113074000	0.136453000
H	-1.392852000	1.503251000	-1.283720000
H	-1.173258000	2.364748000	0.246514000
C	2.413859000	-0.752529000	-0.192473000
C	2.424091000	0.678869000	0.309734000
C	1.198044000	1.392422000	-0.229086000
H	3.228277000	-1.329712000	0.247518000
H	2.540504000	-0.763271000	-1.283322000
H	2.409299000	0.686673000	1.403342000
H	3.325446000	1.193868000	-0.027251000
H	1.098478000	2.384397000	0.217516000
H	1.290673000	1.526911000	-1.316764000
N	1.155441000	-1.374296000	0.206916000
H	1.073076000	-2.359921000	-0.004587000

TMS-SMe

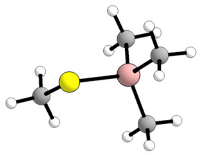

# of imaginary frequencies: 0

E = -847.340460328

C	2.382609000	0.496840000	0.002295000
S	1.255006000	-0.947663000	0.013992000
H	3.398390000	0.115648000	0.102494000
H	2.301413000	1.042350000	-0.937393000
H	2.173427000	1.162238000	0.840114000
Si	-0.665300000	0.066313000	-0.001893000
C	-0.738237000	1.237841000	-1.459817000
H	-1.741290000	1.670319000	-1.538568000
H	-0.029188000	2.063025000	-1.349471000
H	-0.519083000	0.719096000	-2.397022000
C	-1.917193000	-1.311919000	-0.159793000
H	-1.820802000	-2.034739000	0.655127000
H	-2.932759000	-0.905181000	-0.126908000
H	-1.798624000	-1.845914000	-1.106716000
C	-0.888464000	1.012181000	1.596804000
H	-1.852176000	1.532320000	1.591703000
H	-0.869508000	0.339462000	2.458467000
H	-0.107984000	1.765967000	1.733859000

TS1

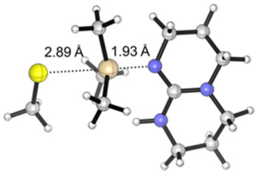

# of imaginary frequencies: 1

E = -1286.10893402

C	-4.259045000	-1.713261000	-0.606819000
S	-4.184036000	-0.031963000	0.116203000
Si	-1.339496000	0.497555000	0.139909000
N	0.556932000	0.837866000	0.066346000
C	-1.489273000	-0.209161000	-1.604319000
C	-1.937385000	2.237855000	0.513421000
H	-1.693767000	-1.283508000	-1.579214000
H	-2.322273000	0.263096000	-2.127306000
H	-0.572771000	-0.051305000	-2.179631000
H	-2.116105000	2.811548000	-0.400446000
H	-2.884083000	2.171355000	1.050515000
H	-1.234071000	2.796499000	1.134657000
C	-1.387588000	-0.640831000	1.645201000
H	-1.735446000	-1.646898000	1.391599000
H	-0.418623000	-0.723178000	2.145861000
H	-2.109490000	-0.227445000	2.351662000
N	2.810674000	0.120908000	0.059374000
C	1.482134000	-0.125422000	0.064513000
H	-5.241709000	-2.164368000	-0.447950000
H	-3.515226000	-2.374230000	-0.150198000

H	-4.074854000	-1.694000000	-1.684290000
N	1.083232000	-1.417283000	0.026417000
C	1.958244000	-2.569502000	0.223313000
H	1.500281000	-3.416982000	-0.286123000
H	2.034390000	-2.808304000	1.289510000
C	3.325412000	-2.250204000	-0.344727000
H	3.266677000	-2.159410000	-1.432477000
H	4.030276000	-3.045568000	-0.100145000
C	3.800694000	-0.943071000	0.258141000
H	4.724713000	-0.612895000	-0.217344000
H	3.995739000	-1.064339000	1.330063000
C	0.978346000	2.242077000	-0.115302000
H	0.254568000	2.729311000	-0.770237000
H	0.948286000	2.755775000	0.850380000
C	2.366722000	2.364864000	-0.707455000
H	2.686321000	3.407354000	-0.672139000
H	2.370512000	2.036219000	-1.750555000
C	3.306375000	1.494543000	0.096555000
H	4.309036000	1.488683000	-0.331206000
H	3.377378000	1.847060000	1.132331000
H	0.094086000	-1.606603000	0.034613000

INT1 = TBD-TMS---SMe + C_6F_6

TBD-TMS---SMe

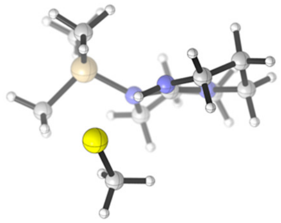

# of imaginary frequencies: 0

E = -1286.12592283

Si	1.545128000	-1.232809000	0.586518000
C	1.361034000	-3.032925000	1.079545000
H	2.256608000	-3.334563000	1.633948000
H	0.501630000	-3.177353000	1.740859000
H	1.261944000	-3.713779000	0.230350000
C	3.002569000	-1.028096000	-0.557364000
H	3.904984000	-1.438766000	-0.092646000
H	2.846955000	-1.557278000	-1.502468000
H	3.175273000	0.031830000	-0.765357000
C	1.690631000	-0.233290000	2.152332000
H	2.510654000	-0.669240000	2.734402000
H	1.925299000	0.815252000	1.963670000
H	0.784739000	-0.296482000	2.761363000
H	0.418224000	1.526473000	0.437944000
N	-0.551788000	1.192333000	0.605628000
C	-0.911954000	0.058479000	0.003496000
C	-1.484764000	2.219738000	1.061104000
N	-2.199714000	-0.199682000	-0.254098000
N	0.062350000	-0.818852000	-0.352359000

H	-1.112304000	2.622001000	2.005127000
H	-1.501196000	3.033361000	0.328642000
C	-2.873970000	1.631773000	1.219705000
C	-2.691323000	-1.454176000	-0.840056000
C	-3.212872000	0.838937000	-0.030168000
C	-0.323493000	-1.789685000	-1.393505000
H	-2.914343000	0.972787000	2.090894000
H	-3.600588000	2.432218000	1.363124000
C	-1.594780000	-2.496644000	-0.960880000
H	-3.097687000	-1.214494000	-1.828101000
H	-3.513309000	-1.811179000	-0.215599000
H	-4.172003000	0.332171000	0.075547000
H	-3.265504000	1.492569000	-0.907331000
H	0.496002000	-2.491935000	-1.542975000
H	-0.477806000	-1.262718000	-2.340809000
H	-1.419464000	-2.989374000	-0.001115000
H	-1.897514000	-3.252612000	-1.687048000
S	2.102295000	2.690468000	-0.378556000
C	1.455152000	2.114187000	-1.997964000
H	1.950537000	2.629933000	-2.824542000
H	1.615376000	1.039444000	-2.134332000
H	0.381667000	2.307195000	-2.089578000

INT2

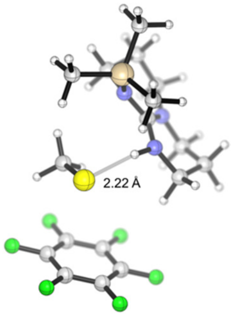

# of imaginary frequencies: 0

E = -2113.73370653

Si	-3.420770000	-1.690087000	0.601276000
C	-5.292622000	-1.572406000	0.631042000
H	-5.696419000	-2.444937000	1.155702000
H	-5.622910000	-0.679727000	1.170672000
H	-5.737074000	-1.555713000	-0.367767000
C	-2.893735000	-3.224346000	-0.316191000
H	-3.352087000	-4.104333000	0.147375000
H	-3.209419000	-3.200877000	-1.363454000
H	-1.806950000	-3.337589000	-0.270742000
C	-2.805434000	-1.663390000	2.361108000
H	-3.312224000	-2.477312000	2.892466000
H	-1.729809000	-1.835391000	2.425054000
H	-3.052180000	-0.730742000	2.874701000
H	-0.793296000	-0.312965000	1.039156000
N	-1.240836000	0.623930000	1.040929000
C	-2.194313000	0.821833000	0.129313000
C	-0.472377000	1.687948000	1.679698000
N	-2.467083000	2.052374000	-0.320032000

N	-2.860205000	-0.261950000	-0.346245000
H	-0.332838000	1.426950000	2.730682000
H	0.514932000	1.741323000	1.210164000
C	-1.193667000	3.013603000	1.528667000
C	-3.530459000	2.365274000	-1.284123000
C	-1.611617000	3.176644000	0.078106000
C	-3.520425000	-0.065194000	-1.649744000
H	-2.082336000	3.039562000	2.165064000
H	-0.535154000	3.830657000	1.825084000
C	-4.409315000	1.162871000	-1.577955000
H	-3.044050000	2.712938000	-2.201269000
H	-4.113672000	3.194444000	-0.877407000
H	-2.192697000	4.087879000	-0.063856000
H	-0.737091000	3.221986000	-0.580345000
H	-4.093592000	-0.959932000	-1.891663000
H	-2.756147000	0.054176000	-2.425141000
H	-5.151553000	1.020087000	-0.788385000
H	-4.935748000	1.325932000	-2.519715000
S	0.613664000	-1.940002000	0.504423000
C	0.240353000	-1.410036000	-1.212897000
H	0.988142000	-1.785085000	-1.916918000
H	-0.733783000	-1.786412000	-1.540745000
H	0.217544000	-0.318290000	-1.297710000
C	2.859157000	1.313093000	-0.615394000

C	2.965746000	0.279213000	-1.529253000
C	3.590398000	-0.899992000	-1.167341000
C	4.114563000	-1.044117000	0.104140000
C	4.020643000	-0.007377000	1.013185000
C	3.389439000	1.170911000	0.655509000
F	2.459242000	0.417907000	-2.750263000
F	3.660844000	-1.907172000	-2.031124000
F	4.683268000	-2.190284000	0.460408000
F	4.512299000	-0.151488000	2.239929000
F	3.297561000	2.167847000	1.530214000
F	2.254724000	2.446186000	-0.961323000

TS2

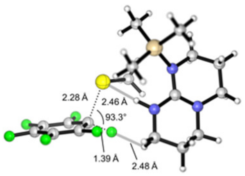

# of imaginary frequencies: 1

E = -2113.71213512

Si	-2.683466000	-2.039136000	0.628050000
C	-4.371053000	-2.509974000	1.291490000
H	-4.285153000	-3.453286000	1.841726000
H	-4.756774000	-1.756415000	1.984273000
H	-5.109113000	-2.662272000	0.499164000
C	-2.094989000	-3.359531000	-0.549893000
H	-2.078124000	-4.319674000	-0.023106000
H	-2.759531000	-3.468541000	-1.411682000

H	-1.083497000	-3.151669000	-0.905725000
C	-1.532528000	-1.799784000	2.074945000
H	-1.544557000	-2.735741000	2.645399000
H	-0.500151000	-1.604063000	1.779850000
H	-1.869496000	-1.004443000	2.744776000
H	-0.711264000	0.197410000	0.511102000
N	-1.409728000	0.929640000	0.653768000
C	-2.584595000	0.738745000	0.043406000
C	-0.956069000	2.191430000	1.242997000
N	-3.371612000	1.783784000	-0.226110000
N	-2.933349000	-0.521105000	-0.318826000
H	-0.570597000	1.986087000	2.242710000
H	-0.137264000	2.580802000	0.634070000
C	-2.096578000	3.192657000	1.295202000
C	-4.719016000	1.682741000	-0.801982000
C	-2.851571000	3.140046000	-0.020273000
C	-3.988045000	-0.597379000	-1.348959000
H	-2.782621000	2.957822000	2.112732000
H	-1.694008000	4.192095000	1.463717000
C	-5.176590000	0.240814000	-0.912250000
H	-4.683895000	2.148669000	-1.791809000
H	-5.393100000	2.270607000	-0.175138000
H	-3.706919000	3.814543000	-0.015432000
H	-2.201359000	3.412052000	-0.858664000

H	-4.267108000	-1.641347000	-1.489400000
H	-3.585625000	-0.232464000	-2.299854000
H	-5.539261000	-0.126537000	0.050909000
H	-5.991503000	0.175388000	-1.634788000
S	0.811554000	-1.115967000	-0.899335000
C	-0.195738000	-0.514190000	-2.295893000
H	0.398722000	-0.451405000	-3.208854000
H	-1.021768000	-1.210038000	-2.466884000
H	-0.609937000	0.471139000	-2.075977000
C	2.330134000	0.530879000	-0.500092000
C	3.473942000	0.327831000	-1.307437000
C	4.672984000	-0.107848000	-0.796331000
C	4.856622000	-0.256086000	0.571126000
C	3.789959000	0.044952000	1.402716000
C	2.591260000	0.485398000	0.888622000
F	3.312227000	0.386011000	-2.644259000
F	5.687384000	-0.375697000	-1.627931000
F	6.050807000	-0.611490000	1.075750000
F	3.935394000	-0.070499000	2.728256000
F	1.560766000	0.703801000	1.731385000
F	1.473601000	1.532787000	-0.927937000

Prod1 = TBD-TMS---F + C_6F_5SMe

TBD-TMS---F

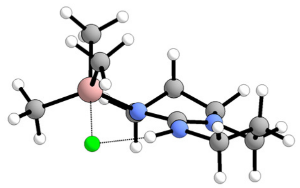

# of imaginary frequencies: 0

E = -947.902087846

Si	1.979151000	-0.304079000	-0.091129000
C	2.350354000	0.964308000	-1.538397000
H	3.279439000	0.647800000	-2.032211000
H	1.565798000	0.958949000	-2.305816000
H	2.505821000	2.005019000	-1.233143000
C	3.430099000	0.192719000	1.025815000
H	4.008991000	-0.685652000	1.326477000
H	4.106794000	0.912151000	0.558458000
H	3.043601000	0.639003000	1.948909000
C	1.968899000	-1.797182000	-1.267563000
H	2.980692000	-1.921341000	-1.672104000
H	1.669472000	-2.743822000	-0.815677000
H	1.319670000	-1.597319000	-2.126412000
F	1.531900000	-1.510096000	1.292536000
H	-0.108679000	-1.866621000	0.559320000
N	-0.927369000	-1.406771000	0.168295000
C	-0.850867000	-0.059986000	0.200770000
C	-2.169013000	-2.171350000	0.095669000
N	-1.976898000	0.667434000	0.082193000

N	0.353227000	0.509180000	0.318620000
H	-1.977712000	-3.072624000	-0.488853000
H	-2.471501000	-2.476298000	1.102761000
C	-3.257802000	-1.326646000	-0.536435000
C	-1.985778000	2.131624000	-0.011193000
C	-3.291397000	0.021563000	0.159875000
C	0.368217000	1.941313000	0.641009000
H	-3.061170000	-1.183583000	-1.602141000
H	-4.221788000	-1.825696000	-0.429443000
C	-0.595745000	2.681703000	-0.266844000
H	-2.386334000	2.532400000	0.926295000
H	-2.673009000	2.404955000	-0.814884000
H	-4.005386000	0.690939000	-0.320429000
H	-3.585144000	-0.091547000	1.209546000
H	1.386805000	2.308980000	0.534541000
H	0.076226000	2.078267000	1.688883000
H	-0.302809000	2.523066000	-1.308063000
H	-0.589508000	3.753653000	-0.063015000

C_6F_5SMe

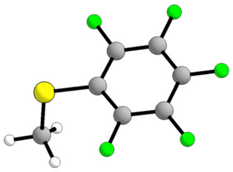

# of imaginary frequencies: 0

E = -1165.86105802

C	1.929204000	0.177709000	0.080013000

C	1.395343000	-1.097281000	0.039113000
C	0.029566000	-1.265035000	-0.112408000
C	-0.833787000	-0.182024000	-0.234186000
C	-0.263766000	1.087322000	-0.200837000
C	1.095822000	1.276852000	-0.037359000
F	2.195522000	-2.153360000	0.158122000
F	-0.439280000	-2.511101000	-0.132324000
F	-1.031874000	2.167775000	-0.330989000
F	1.611058000	2.502947000	-0.008910000
F	3.236634000	0.347488000	0.228647000
S	-2.577222000	-0.414977000	-0.449280000
C	-3.178086000	0.340147000	1.096583000
H	-4.256581000	0.179037000	1.104841000
H	-2.730609000	-0.157964000	1.954956000
H	-2.971577000	1.408680000	1.112247000

TS3

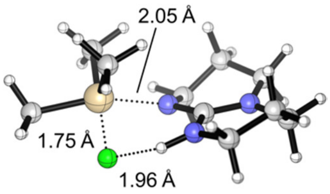

# of imaginary frequencies: 1

E = -947.893707247

Si	-2.084667000	-0.194822000	0.037913000
C	-2.600699000	1.492277000	0.795569000
H	-3.386762000	1.249534000	1.518733000
H	-1.801285000	2.014110000	1.329383000

H	-3.048391000	2.190912000	0.080613000
C	-3.914047000	-0.721428000	-0.331818000
H	-3.993799000	-1.793189000	-0.543638000
H	-4.582513000	-0.490021000	0.504122000
H	-4.304070000	-0.192656000	-1.210924000
C	-1.607453000	-1.289887000	1.499053000
H	-2.439428000	-1.282614000	2.211996000
H	-1.444470000	-2.331536000	1.205207000
H	-0.714420000	-0.943089000	2.025332000
F	-1.666749000	-1.090592000	-1.401630000
H	0.002295000	-1.807820000	-0.656179000
N	0.859332000	-1.413158000	-0.294587000
C	0.890758000	-0.062054000	-0.261597000
C	1.971354000	-2.317871000	-0.025040000
N	2.096366000	0.536937000	-0.119743000
N	-0.246883000	0.618670000	-0.355389000
H	1.620454000	-3.113441000	0.636333000
H	2.300411000	-2.776805000	-0.962765000
C	3.121247000	-1.552095000	0.602936000
C	2.263859000	1.976874000	0.093530000
C	3.318909000	-0.264633000	-0.178558000
C	-0.065953000	2.036402000	-0.678190000
H	2.895864000	-1.307286000	1.644666000
H	4.026456000	-2.160174000	0.579993000

C	0.925170000	2.666774000	0.288286000
H	2.778889000	2.392415000	-0.779465000
H	2.913517000	2.116853000	0.961767000
H	4.121281000	0.335565000	0.250185000
H	3.577912000	-0.482606000	-1.221246000
H	-1.028568000	2.535944000	-0.641071000
H	0.309956000	2.141809000	-1.704191000
H	0.561776000	2.526429000	1.310262000
H	1.040409000	3.737191000	0.107788000

INT3 = TMSF + TBD + TMS-SMe + C_6F_5SMe

TMSF

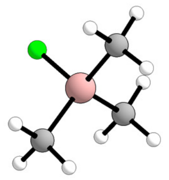

# of imaginary frequencies: 0

E = -509.132824907

Si	0.000287000	-0.000746000	0.002182000
C	-0.472799000	1.719810000	-0.515269000
H	-0.491424000	1.795852000	-1.606802000
H	-1.467127000	1.985542000	-0.145430000
H	0.241325000	2.457788000	-0.139318000
C	1.725712000	-0.451678000	-0.516489000
H	2.008651000	-1.440367000	-0.144780000
H	1.798965000	-0.471562000	-1.608287000
H	2.452953000	0.275758000	-0.144901000

C	-1.255737000	-1.268954000	-0.512646000
H	-1.311933000	-1.323918000	-1.604261000
H	-0.990356000	-2.262259000	-0.140292000
H	-2.251435000	-1.014852000	-0.138382000
F	0.002590000	0.001488000	1.656482000

TS4 = TS1

INT4 = TBD-TMS---SMe + C_6F_5SMe

INT5

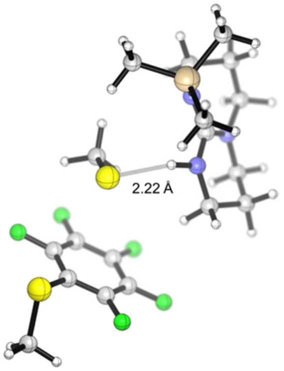

# of imaginary frequencies: 0

E = -2451.99587779

Si	3.672425000	-1.980336000	-0.466293000
C	5.521119000	-2.139301000	-0.736289000
H	5.723996000	-3.121141000	-1.178115000
H	5.888896000	-1.382006000	-1.434893000
H	6.104158000	-2.071809000	0.185659000
C	3.084497000	-3.260550000	0.754026000
H	3.370156000	-4.262930000	0.418794000
H	3.525730000	-3.103168000	1.743154000
H	1.994399000	-3.222174000	0.838445000

C	2.843713000	-2.108353000	-2.131146000
H	3.192711000	-3.039748000	-2.591889000
H	1.755664000	-2.150557000	-2.063190000
H	3.130394000	-1.287010000	-2.793236000
H	1.151169000	-0.444934000	-0.760446000
N	1.638526000	0.455360000	-0.931501000
C	2.743260000	0.693740000	-0.222389000
C	0.801807000	1.496007000	-1.521048000
N	3.162447000	1.945865000	-0.008336000
N	3.416081000	-0.366006000	0.296094000
H	0.405465000	1.120960000	-2.466395000
H	-0.042035000	1.689627000	-0.850699000
C	1.610439000	2.763435000	-1.714132000
C	4.406022000	2.294307000	0.692163000
C	2.324385000	3.081951000	-0.412688000
C	4.307597000	-0.045853000	1.426329000
H	2.347464000	2.629161000	-2.510416000
H	0.951260000	3.587179000	-1.990370000
C	5.247097000	1.071446000	1.013201000
H	4.124439000	2.813722000	1.613850000
H	4.953684000	2.999481000	0.062991000
H	2.985491000	3.941043000	-0.526925000
H	1.606047000	3.304603000	0.383963000
H	4.853399000	-0.945279000	1.709080000

H	3.703526000	0.255508000	2.288530000
H	5.819036000	0.750376000	0.138538000
H	5.947531000	1.317722000	1.812553000
S	-0.404622000	-1.847395000	-0.027346000
C	-0.011855000	-1.034443000	1.570729000
H	-0.745663000	-1.293469000	2.339030000
H	0.972863000	-1.339929000	1.942447000
H	-0.006542000	0.056435000	1.473463000
C	-2.451769000	1.786409000	0.787379000
C	-2.692793000	0.768677000	1.691902000
C	-3.374211000	-0.364169000	1.284307000
C	-3.845404000	-0.508871000	-0.014757000
C	-3.587760000	0.531007000	-0.899888000
C	-2.903675000	1.669356000	-0.515896000
F	-2.261774000	0.885560000	2.946886000
F	-3.555980000	-1.328444000	2.185155000
F	-3.979267000	0.442546000	-2.171937000
F	-2.663743000	2.644903000	-1.390911000
F	-1.782665000	2.870486000	1.166461000
S	-4.650248000	-1.995041000	-0.540949000
C	-6.302167000	-1.329106000	-0.927829000
H	-6.904197000	-2.185539000	-1.233938000
H	-6.743368000	-0.875818000	-0.041668000
H	-6.252023000	-0.612750000	-1.746019000

TS5

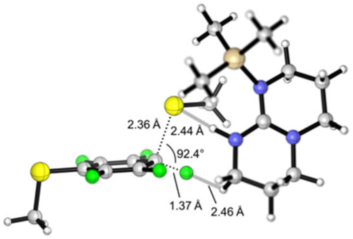

# of imaginary frequencies: 1

E = -2451.97852407

Si	3.126929000	-2.064968000	-0.644896000
C	4.779904000	-2.550927000	-1.380879000
H	4.669169000	-3.503973000	-1.909486000
H	5.132840000	-1.809731000	-2.103869000
H	5.555222000	-2.688070000	-0.621836000
C	2.613155000	-3.344027000	0.610546000
H	2.566077000	-4.323486000	0.122343000
H	3.328117000	-3.422227000	1.434180000
H	1.624060000	-3.123415000	1.018594000
C	1.898690000	-1.877599000	-2.035291000
H	1.884290000	-2.829716000	-2.577889000
H	0.884876000	-1.676108000	-1.685747000
H	2.194997000	-1.100336000	-2.744360000
H	1.156728000	0.177665000	-0.496780000
N	1.850915000	0.902106000	-0.699689000
C	3.051218000	0.730969000	-0.133329000
C	1.372266000	2.146378000	-1.306967000
N	3.837636000	1.787026000	0.091753000

N	3.424559000	-0.517950000	0.239323000
H	0.956752000	1.913866000	-2.288874000
H	0.570408000	2.547578000	-0.683829000
C	2.504925000	3.152049000	-1.418143000
C	5.210717000	1.707255000	0.606702000
C	3.297821000	3.133485000	-0.124043000
C	4.519410000	-0.566648000	1.228226000
H	3.168152000	2.900739000	-2.249635000
H	2.091932000	4.145122000	-1.598825000
C	5.683436000	0.270935000	0.729939000
H	5.217315000	2.195566000	1.586175000
H	5.852166000	2.284554000	-0.062945000
H	4.144856000	3.817169000	-0.165323000
H	2.667700000	3.413739000	0.726894000
H	4.813180000	-1.605436000	1.377039000
H	4.150710000	-0.186969000	2.187190000
H	6.014443000	-0.115177000	-0.237318000
H	6.525127000	0.227232000	1.422712000
S	-0.290590000	-1.097148000	0.990685000
C	0.762942000	-0.476001000	2.345454000
H	0.214493000	-0.453384000	3.288831000
H	1.631362000	-1.130051000	2.463443000
H	1.116282000	0.532732000	2.123103000
C	-1.864644000	0.622713000	0.646119000

C	-2.937619000	0.425544000	1.539920000
C	-4.146240000	-0.067836000	1.120748000
C	-4.445548000	-0.296803000	-0.226987000
C	-3.417211000	0.014919000	-1.121230000
C	-2.203298000	0.512750000	-0.717903000
F	-2.689509000	0.575771000	2.855549000
F	-5.069350000	-0.321339000	2.059688000
F	-3.607040000	-0.150213000	-2.438029000
F	-1.237743000	0.752221000	-1.627560000
F	-0.964332000	1.595089000	0.987966000
S	-6.005030000	-0.923666000	-0.759841000
C	-6.927531000	0.632176000	-0.996300000
H	-7.927476000	0.355918000	-1.334224000
H	-7.000923000	1.176339000	-0.055266000
H	-6.444296000	1.244428000	-1.756982000

Prod2 = TBD-TMS---F + $C_6F_4(SMe)_2$

$C_6F_4(SMe)_2$

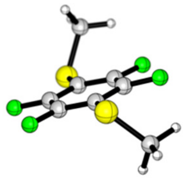

# of imaginary frequencies: 0

E = -1504.11316312

C	0.687233000	-1.335143000	0.093636000
C	-0.687154000	-1.335177000	-0.093588000
C	-1.408876000	-0.150064000	-0.201684000

C	-0.682124000	1.035088000	-0.111995000
C	0.682067000	1.035122000	0.112028000
C	1.408885000	-0.149986000	0.201745000
F	-1.307228000	-2.508202000	-0.177897000
F	-1.302597000	2.209442000	-0.231412000
F	1.302487000	2.209506000	0.231388000
F	1.307345000	-2.508143000	0.177951000
S	-3.151104000	-0.179593000	-0.500494000
C	-3.736941000	0.854715000	0.879932000
H	-3.421596000	1.888051000	0.759717000
H	-4.824241000	0.797019000	0.843657000
H	-3.386956000	0.448035000	1.826980000
S	3.151116000	-0.179313000	0.500619000
C	3.736897000	0.854437000	-0.880257000
H	4.824214000	0.797229000	-0.843668000
H	3.387314000	0.447078000	-1.827161000
H	3.421092000	1.887712000	-0.760701000

TS6 = TS3

Final = TBD + TMSF + $C_6F_4(SMe)_2$

Supplementary References

1. Frisch, M. J.; Trucks, G. W.; Schlegel, H. B.; Scuseria, G. E.; Robb, M. A.; Cheeseman, J. R.; Scalmani, G.; Barone, V.; Mennucci, B.; Petersson, G. A.; Nakatsuji, H.; Caricato, M.; Li, X.; Hratchian, H. P.; Izmaylov, A. F.; Bloino, J.; Zheng, G.; Sonnenberg, J. L.; Hada, M.; Ehara, M.; Toyota, K.; Fukuda, R.; Hasegawa, J.; Ishida, M.; Nakajima, T.; Honda, Y.; Kitao, O.;

Nakai, H.; Vreven, T.; Montgomery, J. A., Jr.; Peralta, J. E.; Ogliaro, F.; Bearpark, M.; Heyd, J. J.; Brothers, E.; Kudin, K. N.; Staroverov, V. N.; Kobayashi, R.; Normand, J.; Raghavachari, K.; Rendell, A.; Burant, J. C.; Iyengar, S. S.; Tomasi, J.; Cossi, M.; Rega, N.; Millam, M. J.; Klene, M.; Knox, J. E.; Cross, J. B.; Bakken, V.; Adamo, C.; Jaramillo, J.; Gomperts, R.; Stratmann, R. E.; Yazyev, O.; Austin, A. J.; Cammi, R.; Pomelli, C.; Ochterski, J. W.; Martin, R. L.; Morokuma, K.; Zakrzewski, V. G.; Voth, G. A.; Salvador, P.; Dannenberg, J. J.; Dapprich, S.; Daniels, A. D.; Farkas, Foresman, J. B.; Ortiz, J. V.; Cioslowski, J.; Fox, D. J. Gaussian, Inc., Wallingford CT, (2009)

2. A. V. Marenich, C. J. Cramer, & D. G. Truhlar, Universal solvation model based on solute electron density and a continuum model of the solvent defined by the bulk dielectric constant and atomic surface tensions. *J. Phys. Chem. B*, **113**, 6378-6396, (2009).

3. Zhao, Y., Truhlar, D. G. The M06 suite of density functionals for main group thermochemistry, thermochemical kinetics, noncovalent interactions, excited states, and transition elements: two new functionals and systematic testing of four M06-class functionals and 12 other functionals Theor. *Chem. Acc.* **120**, 215-241, (2008).

4. Zhao, Y., Truhlar, D. G. Density Functionals with Broad Applicability in Chemistry. *Acc. Chem. Res.* **41**, 157-167, (2008).

5. Weinhold, F., Landis, C. R., Glendening, E. D. What is NBO analysis and how is it useful? *International Reviews in Physical Chemistry*, **35**, 3, 399-440, (2016).

6. Reed, A. E., Weinhold, F. Natural localized molecular orbitals. *J. Chem. Phys.* **83**, 1736-1740, (1985).

7. Reed, A. E., Weinhold, F. Natural Bond Orbital Analysis of Internal Rotation Barriers and Related Phenomena. *Isr. J. Chem.* **31**, 277-285, (1991).

8. Reed, A. E., Curtiss, L. A., Intermolecular interactions from a natural bond orbital, donor-acceptor viewpoint. Weinhold, F. *Chem. Rev.* **88**, 899-926, (1988).

9. Weinhold, F. Natural bond orbital methods. in Schleyer P.v.R., Allinger, N. L., Clark. T. (eds) *Encyclopedia of Computational Chemistry*: Wiley: New-York, **3**, 1792-1811, (1998).

10. CYLview, 1.0b; Legault, C. Y., Université de Sherbrooke, 2009 (http://www.cylview.org)
