# Supplementary material for: Organocatalyzed synthesis of fluorinated poly(aryl thioethers)
Source: Nat Commun. 2017 Aug 1;8:166. doi: 10.1038/s41467-017-00186-3 (PMC5537313; doi:10.1038/s41467-017-00186-3)
Supplement: Supplementary file 2 — Peer Review [file 41467_2017_186_MOESM2_ESM.pdf]

Reviewers' comments:

Reviewer #1 (Remarks to the Author):

The authors present a new polymerization method towards fluorinated poly(aryl thioether)s using organocatalyzed nucleophilic aromatic substitution of silly protected dithiols. The proposed chemistry is novel and a much needed addition to the synthetic polymerization tool box as these polymers are highly desirable for a wide range of high value applications, including coatings, microelectronics, gas separation membranes just to name a few. The proof-of-principle experiments are convincing in that this is useful and scaleable chemistry that can easily be adapted by researchers in the filed or by industry. A very important advantage is the fact that the chemistry can be performed at room temperature, only small amounts of catalyst is needed, reaction times are short and clean-up procedures can be kept to a minimum (not salts!). I have very little experience with computational studies so I cannot comment on this part of the study. In conclusion, this paper will open a new synthetic route towards the design and synthesis of new high-performance fluorinated polymers and can be published as is.

Reviewer #2 (Remarks to the Author):

Journal: Nature Communications,
Title: "Organocatalyzed Synthesis of Fluorinated Poly(aryl thioethers)"

This article describes a highly efficient new route to fluorinated poly(aryl thioethers) via an organocatalyzed nucleophilic aromatic substitution of silyl-protected dithiols. The corresponding polymers were successfully prepared rapidly at room temperature with a low catalyst loading. Thus, the reviewer recommends the manuscript to be published in this journal. For the benefit of the readers, the following points need clarifying.
1. As silyl-protected dithiols in place of dithiols were employed as a nucleophile, a catalytic amount of base is enough to proceed the ANAr. However, this polymerization is not an atom economical one due to producing a large amount of by-product, trimethylsilyl fluoride. Authors should answer this comment.
2. Disilyl monomer from 4,4'-thiobisbenzenethiol is unstable due to higher acidity of aromatic thiol compared to aliphatic thiol. Thus, to emphasis the effectiveness of this method, the results of polymerization using disilyl monomer should compare to that of polymerization between 4,4'-thiobisbenzenethiol and hexafluorobenzene or decafluorobiphenyl in the presence of stoichiometric base.
3. Line 107 and 108. Authors described as follows. When decafluorobiphenyl was utilized as a monomer with 2a, no catalyst was necessary to initiate polymerization. Where is the data?
4. Line 108 and 109. Authors described as follows. Dissolution of both monomers in DMF was sufficient to induce rapid polymerization to afford 2c (Figure 4, entry 3). Did you observe the formation of trimethylsilyl fluoride?
5. Figures 2 and 4. The yields of all polymers should be added.

Reviewer #3 (Remarks to the Author):

The authors report the synthesis of fluorinated poly(aryl thioethers) via a catalytic process rather than stoichiometric consumption of base in a typical SNAr process. In their process, salts are not formed which can be problematic in certain applications because TMSF can be easily eliminated. The concept presented by the authors are interesting but the novelty is reduced by the fact that silylation was previously applied to synthesis of aromatic poly (ether ketone)s from silylated bisphenols, which concept is close to silylated thiols, as referenced by the author in ref. 28.

Questions to be addressed:
• In Figure 2, reaction time seems rather arbitrary. Can the authors the quantify consumption of C6F6 and thus provide the rationale for the chosen reaction time?
• How the new synthesis route compared to the conventional route in terms of differences inn properties for the synthesized poly(aryl thioethers)?
• In the present manuscript, there is no systematic trend in terms of how polymer properties change as a function of base loading and types of base, and the property changes quite drastically in strictly anhydrous and oxygen free environment. In order to demonstrate utility and generality of this catalysis, the authors should demonstrate control or establish trend of how polymer properties (e.g. Tg or Tm) evolve as a function of process variables (base loading, types of base, solvent, temperature, presence of O2 and etc).
• To establish generality and utility of this methodology, the scope of the nucleophile and electrophile should be expanded. The current scope shown in Figure 4 is too limited in my opinion.
• The catalysts used in the manuscript are just bases, not custom designed organic catalyst for chemical transformation, thus the word choice of “organocatalyst” may not be appropriate.
• After TMS binds to N of TBD, the N-H should become protic, how acidic is that N-H proton? The pKa and thus the acidity of the proton should be readily computed using DFT. Is the N-H proton acidic enough to protonate MeS-?
• The authors suggest that the C-H proton should be protic enough to aid the F- dissociation and the C-H was determined to have a partial positive charge of 0.21. Can the authors report the partial charge of other protons on TBD at least in the supporting information?
• At TS2, The F-H (H from the C-H) distance is 2.48A, which is quite far compared to standard H-bonding distance of <2A, and should not exert much stabilization to the dissociating F-. What is the F-H distance, where H is the N-H? This H should have the most protic character, and the Prod1 also shows that the product is where F- is coordinated to the N-H. TS-2, currently shown, may not be the only unique TS, where a lower energy TS could be found where F- is H-bond to the N-H at the transition state.
• H-bond to MeS- should lower nucleophilicity of the MeS-, did the authors examine this? What about examining 7-Methyl-1,5,7-triazabicyclo[4.4.0]dec-5-ene or MTBD, where there is no N-H?
• Overall, my impression is that the DFT mechanism drawn from TBD is very specific and not general enough to explain catalysis performed using other bases e.g. DBU base, which doesn’t have two participating N’s in the mechanism. Also, tetrabutylammonium fluoride (Figure 2), which doesn’t have basic functionality and yet still catalyzes the synthesis of poly(aryl thioethers), which raises concern on the validity of the DFT mechanism presented.

Reviewer #2

1. As silyl-protected dithiols in place of dithiols were employed as a nucleophile, a catalytic amount of base is enough to proceed the ANAr. However, this polymerization is not an atom economical one due to producing a large amount of by-product, trimethylsilyl fluoride. Authors should answer this comment.

Response: As the reaction is a condensation polymerization proceeding via nucleophilic aromatic substitution, there will always be a stoichiometric amount of byproduct regardless of the type of nucleophile used. Under typical S_NAr reactions using dithiols with stoichiometric base, a corresponding stoichiometric amount of fluoride salt will be generated as a byproduct. The advantage of using TMS-protected dithiols is that the TMSF byproduct is a gas and can easily be separated from the polymer as opposed to fluoride salts, which can be more difficult to remove.

2. Disilyl monomer from 4,4'-thiobisbenzenethiol is unstable due to higher acidity of aromatic thiol compared to aliphatic thiol. Thus, to emphasis the effectiveness of this method, the results of polymerization using disilyl monomer should compare to that of polymerization between 4,4'-thiobisbenzenethiol and hexafluorobenzene or decafluorobiphenyl in the presence of stoichiometric base.

Response: The reaction between 4,4'-thiobisbenzenethiol and decafluorobiphenyl under stoichiometric base conditions provided a polymer with a similar broad distribution of molecular weights, although somewhat lower than those using our conditions (see Supporting Information). It is anticipated that further optimization of the stoichiometric base conditions could yield a polymer with similar molecular weights as those observed using the disilyl protected monitor. However, despite the lower stability of the disilyl protected monomer, the speed of the polymerization and ease of workup make our method an efficient means of accessing these polymers and highly complementary to traditional approaches.

3. Line 107 and 108. Authors described as follows. When decafluorobiphenyl was utilized as a monomer with 2a, no catalyst was necessary to initiate polymerization. Where is the data?

Response: The data is presented in Figure 4, entry 3. The wording of these sentences has been updated to improve the clarity for the reader.

4. Line 108 and 109. Authors described as follows. Dissolution of both monomers in DMF was sufficient to induce rapid polymerization to afford 2c (Figure 4, entry 3). Did you observe the formation of trimethylsilyl fluoride?

Response: The evolution of TMSF gas was observed during the course of the reaction.

5. Figures 2 and 4. The yields of all polymers should be added.

Response: As this reaction is a step-growth polymerization, the conversions of the monomers is very high and rapid as revealed by early time course experiments (data added to Supporting Information). With these data in mind, Figure 2 is meant to illustrate the influence of catalyst identity and loading on the molecular weight distribution. The main exceptions are the reactions catalyzed by weaker amine bases which did not proceed to full conversion (Figure 2 has been updated to indicate the conversion in these cases). Figure 4 has been updated with illustrative yields based on recovered material for the different examples.

Reviewer #3

1. In Figure 2, reaction time seems rather arbitrary. Can the authors the quantify consumption of C6F6 and thus provide the rationale for the chosen reaction time?

Response: Early time course experiments at higher catalyst loadings revealed rapid consumption of C_6F_6 (full conversion in less than 1 minute). As such, for most cases we selected a reaction time between 5 and 15 minutes during which time the polymers had precipitated out of solution. In cases where weak bases were used as catalysts, longer reaction times where necessary to observe polymer formation. Data for these time course experiments have been added to the supporting information.

2. How the new synthesis route compared to the conventional route in terms of differences in properties for the synthesized poly(aryl thioethers)?

Response: Utilizing stoichiometric base and longer reaction times typical of conventional S_NAr polymerizations gave polymers with similar properties (This data has been added to the Supporting Information). These results are expected as polymer physical properties such as T_g have a much stronger dependence on the nature of the monomers as opposed to molecular weight distribution.

3. In the present manuscript, there is no systematic trend in terms of how polymer properties change as a function of base loading and types of base, and the property changes quite drastically in strictly anhydrous and oxygen free environment. In order to demonstrate utility and generality of this catalysis, the authors should demonstrate control or establish trend of how polymer properties (e.g. Tg or Tm) evolve as a function of process variables (base loading, types of base, solvent, temperature, presence of O2 and etc).

Response: During the course of our study we observed no significant change in polymer properties (T_g or T_m) based on catalyst loading, solvent, catalyst identity, or atmosphere. This is not unexpected as polymer physical properties are more strongly dependent on the identity of the monomer as opposed to the polymerization conditions.

4. To establish generality and utility of this methodology, the scope of the nucleophile and electrophile should be expanded. The current scope shown in Figure 4 is too limited in my opinion.

Response: While the substrate scope can certainly be expanded in both terms of electrophile and nucleophile, for the purposes of this work we feel that the current scope adequately showcases the methodology for many of the common nucleophile types (aryl and alkyl thiols) and commercially available perfluoroarenes or highly activated fluoroaromatics. Future work will focus on tailoring the monomers for specific applications and desired polymer properties.

5. The catalysts used in the manuscript are just bases, not custom designed organic catalyst for chemical transformation, thus the word choice of "organocatalyst" may not be appropriate.

Response: Our choice of the term of organocatalyst comes for TBD and DBU being from a long and well-established history of using these bases as highly effective catalysts for ring-opening polymerization. This is one of many examples of using these bases as catalysts in organic transformations. Additionally, the computational work in this paper suggests that TBD has a more complex role than simply being a base.

For an example reference on the prior use of TBD as an organocatalyst see the following:

Pratt, R. C.; Lohmeijer, B. G. G.; Long, D. A.; Waymouth, R. M.; Hedrick, J. L. Triazabicyclodecene: a simple bifunctional organocatalyst for acyl transfer and ring-opening polymerization of cyclic esters. *J. Am. Chem Soc.* **2006**, *128*, 4556-4557.

6. After TMS binds to N of TBD, the N-H should become protic, how acidic is that N-H proton? The pKa and thus the acidity of the proton should be readily computed using DFT. Is the N-H proton acidic enough to protonate MeS-?

Response: We appreciate the question. We attempted to calculate the complex between MeSH and TBD-TMS, in the same fashion we had the $MeS^{-}(TBD\text{-}TMS)^{+}$ complex. The geometry optimization collapsed back to the original $MeS^{-}(TBD\text{-}TMS)^{+}$ complex, suggesting that it is the global minima. We then proceeded to perform a relaxed scan for the S…H distance describing the transfer of H^{+} to the thiolate moiety.

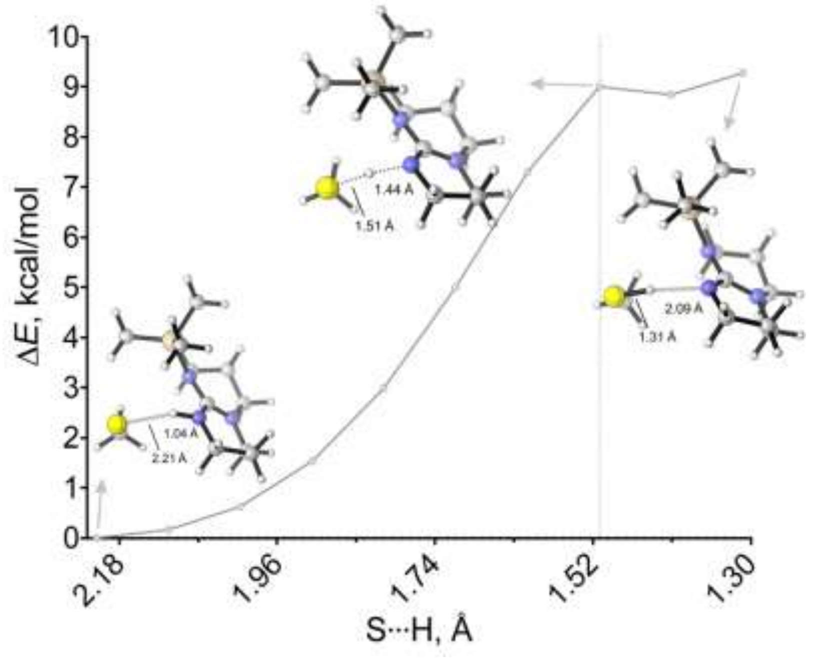

We note that there should be a TS-like point for such transfer, at 9.0 kcal/mol. However, the formed (MeSH)(TBD-TMS) complex lies above 9.3 kcal/mol. In summary, the N-H proton is NOT acidic enough to protonate MeS-.

7. The authors suggest that the C-H proton should be protic enough to aid the F- dissociation and the C-H was determined to have a partial positive charge of 0.21. Can the authors report the partial charge of other protons on TBD at least in the supporting information?

Response: We appreciate the reviewer's question. The data is now present in the SI and also here. TS2:

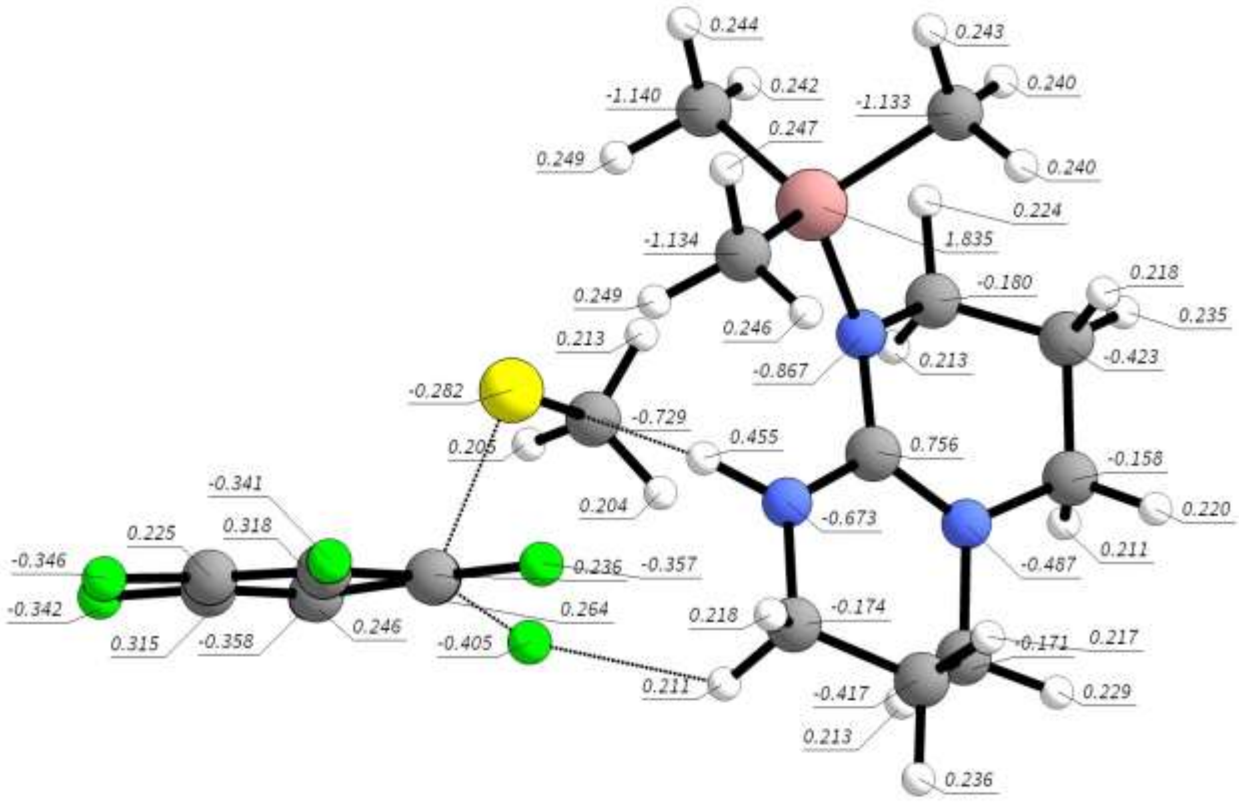

The key finding here is that any C-H in the vicinity of the departing fluoride can provide stabilization to the forming anion. Although other C-H bonds might be slightly more acidic around TBD, the C-H bond in question is the only one in the right position that is able to stabilize the leaving F- group. Thus, this interaction is uniquely capable of taking advantage of synergy with other interactions. It is possible, of course, that after the substitution TS, the departing fluoride can coordinate with a more positively charged hydrogen.

8. At TS2, The F-H (H from the C-H) distance is 2.48A, which is quite far compared to standard H-bonding distance of <2A, and should not exert much stabilization to the dissociating F-. What is the F-H distance, where H is the N-H? This H should have the most protic character, and the Prod1 also shows that the product is where F- is coordinated to the N-H. TS-2, currently shown, may not be the only unique TS, where a lower energy TS could be found where F- is H-bond to the N-H at the transition state.

Response: We appreciate the reviewer's question. Indeed, that N-H bears the most positive Hydrogen atom in TS2: its natural charge is of +0.46 *e*. The F-H (of N-H) bond distance is 2.95Å and, according to NBO analysis, there are no indications of orbital overlap between the two species. However, that same N-H bond stabilizes the MeS- group via H-bonding (S•••H distance is 2.43Å). Due to the geometry of this TS and the necessity of stabilizing the MeS- group prior to the TS, it is unlikely the same N-H bond would simultaneously also stabilize the leaving F- group. One must note the complexity of these transition states: the organocatalyst plays many roles stabilizing both the attacking MeS- group and the leaving F- group. Analysis of the electrostatic interaction between F- and that H (approximated as NBO point charges) suggests that Coulombic attraction accounts for ~2.7 kcal/mol stabilization in TS2 and ~2.5 kcal/mol stabilization in TS5.

9. H-bond to MeS- should lower nucleophilicity of the MeS-, did the authors examine this? What about examining 7-Methyl-1,5,7-triazabicyclo[4.4.0]dec-5-ene or MTBD, where there is no N-H?

Response: Although hydrogen bonding to the thiolate would attenuate its nucleophilicity, our work suggests that the hydrogen bonding is primarily between the N-H and the in-plane lone pair of sulfur (see Figure 6a). This arrangement still leaves the other lone pair capable of nucleophilic attack on the arene ring, which is further assisted by stabilization of the departing fluoride by C-H bonds on the catalyst.

While MTBD was not examined as a catalyst for this communication, it would likely exhibit similar reactivity as DBU, which also lacks an N-H for hydrogen bonding yet is still a viable catalyst for the polymerization. Future work will aim to provide further insight into how structural differences affect the mechanistic pathway of the polymerization reaction.

10. Overall, my impression is that the DFT mechanism drawn from TBD is very specific and not general enough to explain catalysis performed using other bases e.g. DBU base, which doesn't have two participating N's in the mechanism. Also, tetrabutylammonium fluoride (Figure 2), which doesn't have basic functionality and yet still catalyzes the synthesis of poly(aryl thioethers), which raises concern on the validity of the DFT mechanism presented.

Response: The results from the computational investigations into TBD catalyzed process are indeed going to be highly specific to the TBD process and were never meant to be presented as a potential mechanism for all of the catalyzed polymerizations discussed in the paper. Rather, we felt that the insights gained by understanding the TBD mechanism would not only help us understand the high reactivity of TBD in catalyzing the polymerization reactions, but also have applicability to understanding closely related systems such as DBU. Additionally, we feel that the results generated from the study of the TBD system will enable the design of improved catalyst systems for this reaction. A comprehensive analysis of the mechanistic differences between the amine containing catalysts is currently underway and will be reported in due course.

The TBAF result is not surprising despite its lack of basic nitrogen functional groups. Similar polymerizations using TBAF as a catalyst with carbon nucleophiles have been previously reported (see reference 3). In the case of poly(aryl thioethers), it is anticipated that the TBAF catalyzed process goes through a very different mechanism that does not involve the TMS-amine complex formation (such as INT1, Figure 5) as proposed in the TBD catalyzed reaction. Instead, the mechanism likely proceeds in a manner analogous to similar polymerizations reported for carbon nucleophiles (see reference 3). Therefore, the fact that TBAF does catalyze the polymerization does not invalidate the DFT mechanism presented for the TBD catalyzed reaction.

We appreciate these helpful comments.

REVIEWERS' COMMENTS:

Reviewer #2 (Remarks to the Author):

The revised manuscript is now acceptable for publication.

Reviewer #3 (Remarks to the Author):

The authors have adequately addressed my concerns and questions and I support the publication in its current form.
